# Premature Ovarian Insufficiency in Primary Care: A Scoping Review of Recognition, Diagnostic Delay and Interdisciplinary Care Pathways

**DOI:** 10.3390/healthcare14213795

**Published:** 2026-11-06

**Authors:** Iulia Silcău, Ciprian-Nicușor Solomon, Laura Maghiar, Ioana Adelina Faur, Irina Daniela Henegariu, Luana Ghilea (Seles), Cristian Sava, Alin Bodog, Teodor Andrei Maghiar, Carmen Anca Huniadi

**Affiliations:** 1 Doctoral School of Biomedical Sciences, University of Oradea, Universității Str., No. 1, 410087 Oradea, Romania; silcau_iulia@yahoo.com (I.S.); irinahenegariu@gmail.com (I.D.H.); seles.luana@student.uoradea.ro (L.G.); 2 1st Clinic of Obstetrics and Gynecology, “Pius Brînzeu” Clinical County Emergency Hospital Timisoara, Liviu Rebreanu Boulevard, No. 156, 300723 Timisoara, Romania; dr.solomonciprian@protonmail.com; 3 Department of Psycho-Neurosciences and Rehabilitation, Faculty of Medicine and Pharmacy, University of Oradea, Universității Str., No. 1, 410087 Oradea, Romania; 4 Doctoral School of Medicine, “Victor Babeș” University of Medicine and Pharmacy Timisoara, Eftimie Murgu Square 2, 300041 Timisoara, Romania; 5 Residency Program in Obstetrics and Gynecology, Emergency Clinical County Hospital of Arad, Andrenyi Károly Street No. 2–4, 310037 Arad, Romania; 6 Residency Program in Obstetrics and Gynecology, County Clinical Emergency Hospital of Oradea, Gheorghe Doja Street No. 65, 410169 Oradea, Romania; 7 Department of Medical Disciplines, Faculty of Medicine and Pharmacy, University of Oradea, 410073 Oradea, Romania; cristian.sava@didactic.uoradea.ro; 8 Pediatrics Department, Bihor County Clinical Emergency Hospital, 410167 Oradea, Romania; 9 Department of Surgical Sciences, Obstetrics and Gynecology, Faculty of Medicine and Pharmacy, University of Oradea, Universității Str., No. 1, 410087 Oradea, Romania; alin.bodog@didactic.uoradea.ro; 10 Department of Surgery, Pelican Hospital, Corneliu Coposu Str., No. 2, 410450 Oradea, Romania; teodormaghiar@yahoo.com; 11 Department of Surgical Disciplines, Faculty of Medicine and Pharmacy, University of Oradea, Universității Str., No. 1, 410087 Oradea, Romania; ahuniadi@uoradea.ro

**Keywords:** premature ovarian insufficiency, primary ovarian insufficiency, primary health care, general practice, family physician, diagnostic delay, hormone therapy, interdisciplinary care, scoping review

## Abstract

**Background/Objectives**: Premature ovarian insufficiency (POI) affects approximately 3.5% of women with lifelong bone, cardiovascular, psychological and reproductive consequences. International guidance assigns primary care important roles in recognition, diagnosis and follow-up. This review mapped evidence across the POI care continuum. **Methods**: Following Joanna Briggs Institute methodology and PRISMA-ScR, PubMed/MEDLINE, Scopus and Web of Science were searched from inception to 4 October 2026. Two reviewers independently screened records and assessed full texts. Data were charted by one reviewer, verified by a second and synthesised across five domains. **Results**: Of 1327 records (806 after deduplication), 91 full texts were assessed and 53 sources included (31 empirical; 22 secondary/prescriptive). Primary-care guidance was common, but empirical evidence from primary care was limited. Diagnostic delay of months to years was reported, and in one survey more than half of women consulted three or more clinicians before diagnosis. In one physician survey, supported by a smaller pilot survey, primary care physicians initiated hormone therapy less often than specialists, and gaps in long-term follow-up persisted. No formally evaluated shared-care model was identified. **Conclusions**: Primary care is central to recommended POI care, but implementation remains poorly studied. Research should quantify real-world primary care performance and test structured interdisciplinary pathways.

## 1. Introduction

**Figure 1:**
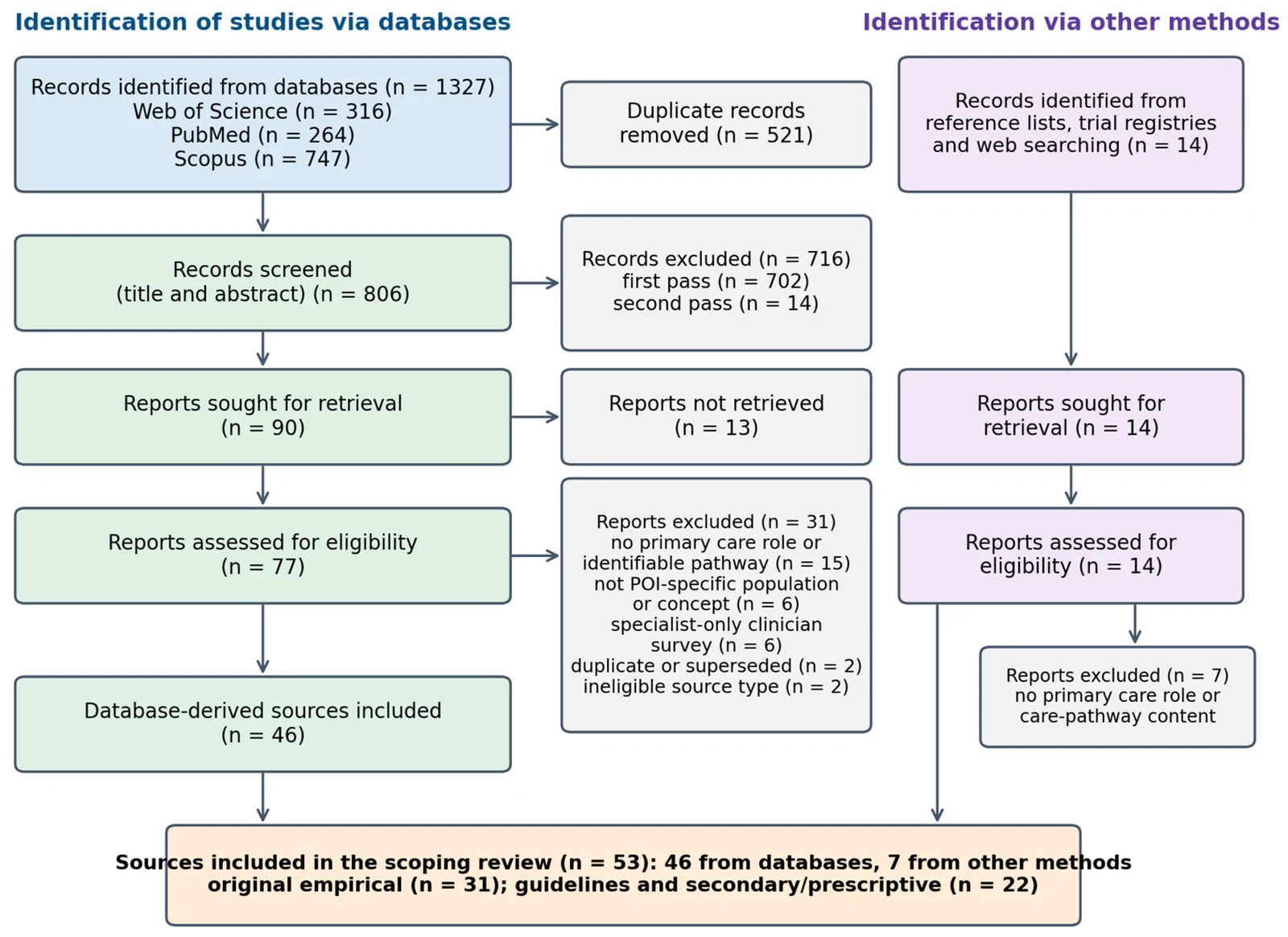
Flow diagram of the identification, screening and inclusion of sources in the scoping review.

**Figure 2:**
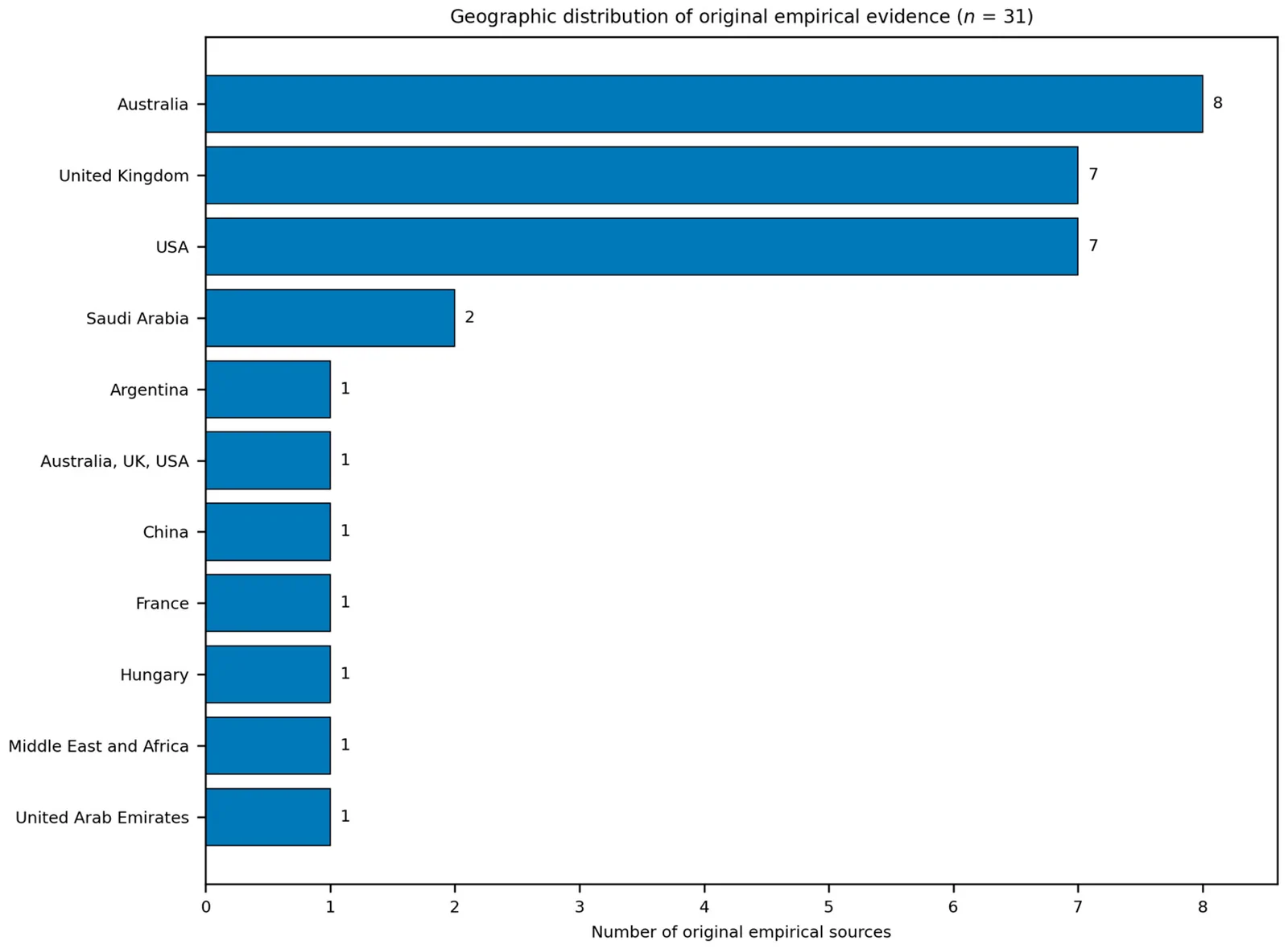
Geographic distribution of original empirical evidence (*n* = 31). Multinational samples are shown separately.

Premature ovarian insufficiency (POI) is the loss of ovarian activity before the age of 40 years, diagnosed on the basis of disordered menstrual cycles for at least four months together with a follicle-stimulating hormone (FSH) concentration above 25 IU/L [1]. The condition was long considered rare, with a prevalence of about 1%, but a systematic review and meta-analysis published in 2023 estimated a pooled global prevalence of 3.5%, and the 2024 international guideline adopted this figure [1,2]. An earlier meta-analysis had already reported a pooled prevalence of 3.7% for POI and 12.2% for early menopause [3]. Population-based data from Finland show a rising incidence and a strong familial component [4]. POI is therefore not an exceptional diagnosis in a general practice population of women of reproductive age.

The consequences of untreated POI are substantial and extend well beyond infertility. Prolonged oestrogen deficiency increases the risk of osteoporosis and fracture, cardiovascular disease, cognitive decline and premature mortality, and the diagnosis itself is associated with high rates of depressive and anxiety symptoms and impaired quality of life [1,5,6]. Women with POI also carry an excess burden of severe autoimmune disease [7]. Hormone therapy (HT), generally continued until the average age of natural menopause, is recommended for women without contraindications to mitigate the consequences of oestrogen deficiency [1,8]. Early diagnosis is therefore a determinant of long-term outcome, not merely of symptom control.

The diagnostic pathway, however, usually begins in primary care. The cardinal presenting symptom is oligo- or amenorrhoea, and most women consult a general practitioner or family physician first [9]. Early studies showed that the diagnosis is frequently missed at this stage: in a cohort evaluated at the National Institutes of Health, more than half of women reported consulting three or more clinicians before POI was identified, and a quarter waited more than five years [10]. Later surveys from Australia and the United States continue to document delays of one to several years [11,12]. The 2024 guideline, developed jointly by ESHRE, ASRM, IMS and CRE-WHiRL, explicitly targets primary care among its users and recommends that healthcare professionals consider and exclude POI in any woman under 40 with irregular cycles, amenorrhoea or symptoms of oestrogen deficiency [1]. A guideline summary written for general practitioners was published in 2026 [9], a dedicated toolkit for the primary care physician had been published earlier [13], and a primary care textbook chapter and an earlier general practice review set out the same recommendations for general practice [14,15].

Despite this clear mandate, the role of primary care in POI has not been systematically examined. The published literature consists largely of narrative reviews and guideline summaries addressed to general practitioners; original data on what actually happens in primary care, how women are recognised, how long diagnosis takes, how referral to gynaecology and endocrinology is organised, and whether long-term follow-up is maintained, are dispersed across patient surveys, registry analyses, physician questionnaires and qualitative studies. A recent physician survey found that specialists initiate HT more than twice as often as primary care physicians, which suggests that the gap between guideline and practice is widest precisely where most women enter the system [16]. No previous review has mapped this body of evidence with the care continuum as its organising frame.

A scoping review is the appropriate design when the aim is to map the extent, range and nature of evidence on a topic, identify the types of evidence available and locate gaps, rather than to estimate a pooled effect [17,18]. We used the care continuum as the organising frame because it mirrors the sequence in which a woman with POI moves through the health system: recognition at first presentation, the interval to diagnosis, referral and shared management with gynaecology or endocrinology, long-term follow-up, and the knowledge and attitudes of the clinicians who provide each of these steps. Mapping evidence onto this sequence makes it possible to distinguish stages for which primary care guidance exists from stages for which primary care practice has actually been measured. The present review therefore aims to map the evidence on five stages of POI care in primary care: recognition and initial evaluation; the magnitude, determinants and consequences of diagnostic delay; referral and interdisciplinary care pathways between primary care, gynaecology and other specialties; long-term follow-up, including HT adherence and chronic disease surveillance; and the knowledge, attitudes and practices of family physicians. The results are intended to inform both clinical practice and the design of original studies on integrated care for POI.

## 2. Materials and Methods

### 2.1. Design and Protocol

This scoping review was conducted according to the Joanna Briggs Institute (JBI) methodology for scoping reviews, which builds on the framework of Arksey and O’Malley, and is reported in accordance with the Preferred Reporting Items for Systematic Reviews and Meta-Analyses extension for Scoping Reviews (PRISMA-ScR) [17,18,19]; the completed PRISMA-ScR checklist is provided as File S1. The protocol was registered on the Open Science Framework (OSF; https://osf.io/erf27 (accessed on 6 October 2026); DOI: 10.17605/OSF.IO/ERF27). An update dated 27 October 2026 amended the data-sharing timetable so that the retained review materials will be made openly available upon publication of the article rather than at manuscript submission; this change did not alter the eligibility criteria, screening decisions, included sources or synthesis. Database searching and title/abstract screening had commenced before registration, whereas full-text eligibility assessment, data charting and evidence synthesis had not; accordingly, this is a protocol registration rather than a preregistration. Because scoping reviews map evidence rather than appraise it for effect estimation, no formal risk-of-bias assessment was performed, in line with JBI guidance [18].

### 2.2. Review Questions and Eligibility Criteria

Eligibility was defined using the Population, Concept, Context (PCC) framework (Table 1).

Reviews and guideline summaries were included deliberately, since one aim was to characterise the balance between the prescriptive care-pathway literature and original data generated in, or interfacing with, primary care. For specialist-setting empirical studies, context eligibility required an identifiable care-pathway outcome relevant to the primary-specialist interface (for example, diagnostic delay, referral, treatment continuity or follow-up); these sources were coded as ‘Not studied directly’ rather than as direct primary-care evidence. In secondary/prescriptive sources, ‘Not applicable’ denoted that primary care was not an empirical variable and did not itself establish eligibility.

### 2.3. Information Sources and Search Strategy

PubMed/MEDLINE, Scopus and Web of Science Core Collection were searched on 4 October 2026. The PubMed strategy combined controlled vocabulary and free-text terms for the condition and for the setting:

(“Primary Ovarian Insufficiency”[Mesh] OR “premature ovarian insufficiency”[tiab] OR “primary ovarian insufficiency”[tiab] OR “premature ovarian failure”[tiab] OR “premature menopause”[tiab] OR “early menopause”[tiab] OR “hypergonadotropic amenorrh*”[tiab])

AND

(“Primary Health Care”[Mesh] OR “General Practice”[Mesh] OR “General Practitioners”[Mesh] OR “Physicians, Family”[Mesh] OR “Physicians, Primary Care”[Mesh] OR “Family Practice”[Mesh] OR “Referral and Consultation”[Mesh] OR “Delayed Diagnosis”[Mesh] OR “primary care”[tiab] OR “general practi*”[tiab] OR “family physician*”[tiab] OR “family doctor*”[tiab] OR “family medicine”[tiab] OR “diagnostic delay”[tiab] OR “delayed diagnosis”[tiab] OR “time to diagnosis”[tiab] OR referral[tiab] OR “care pathway*”[tiab] OR “shared care”[tiab] OR “interdisciplinary”[tiab] OR “multidisciplinary”[tiab] OR “knowledge, attitudes”[tiab] OR “physician survey”[tiab])

The strategy was adapted for Scopus (TITLE-ABS-KEY fields; English language and Article or Review document types applied during screening) and for Web of Science (Topic field was refined to English, Article and Review Article) using the same free-text terms; PubMed was limited to Humans and English. The complete search strategies for all three databases, with search dates, filters and numbers of records retrieved, are provided in Table S1. The reference lists of all included studies and of the 2016 and 2024 ESHRE guidelines were hand-searched. The grey literature was limited to guideline documents and toolkits published by professional societies (ESHRE, ASRM, IMS, British Menopause Society, RACGP), identified through society websites. Clinical trial registries (ClinicalTrials.gov) were screened for registered studies on POI diagnosis and follow-up practices.

### 2.4. Study Selection

Records were exported to a structured Microsoft Excel screening workbook and duplicates were removed using DOI, PMID and normalised title. Two reviewers (I.S. and L.M.) independently screened titles and abstracts and subsequently assessed full texts against the eligibility criteria. AI tools were used for a preliminary, non-decisional survey and organisation of the literature records retrieved from the searched databases and as a supportive aid for provisional record classification; all eligibility decisions were made by the two human reviewers, with final decisions recorded in the screening workbook. At the title and abstract stage, the two human reviewers (I.S., L.M.) independently screened all 806 records against the predefined eligibility criteria; paired reviewer-level decisions were not retained for this initial screening or for the initial full-text assessments, so agreement was quantified for the corrective second pass and the subsequent full-text set described below. Because an initial closure of 41 records at the abstract stage had not been applied consistently, these records were re-screened in a second pass under explicit decision rules; three were removed administratively (a co-published duplicate, a record already excluded after full-text assessment and a source already included). Although the registered protocol specified independent dual screening and the registration form specified reporting percentage agreement and Cohen’s κ at each stage, complete paired reviewer-level decisions were not retained for the initial title and abstract screening set. Inter-reviewer agreement was therefore quantified for the second-pass screening and for the subsequent corrective full-text assessment set. For the 38 records assessed in the second pass, reviewers, each blinded to the other’s decisions, agreed on 35 (92.1%; Cohen’s κ = 0.84); the three disagreements were resolved according to the registered conservative rule whereby any record retained by either reviewer proceeded to full-text assessment. Of the 38 records independently re-screened in the second pass, 14 were initially excluded and 24 proceeded to full-text assessment. One of the 14 (Mittal et al. [20]) was subsequently reconsidered for consistency with the operational “not studied directly” rule, resulting in a corrective full-text set of 25 reports and 13 second-pass exclusions; together with the co-published duplicate removed administratively, 14 records were excluded at the second-pass stage (Figure 1; Table S2A). For these 25 reports, reviewers agreed on 21 (84.0%; Cohen’s κ = 0.68); the four disagreements were resolved by the third reviewer (C.A.H.) acting as arbiter. Reasons for full-text exclusion were recorded for every report (Table S2B); reports that could not be retrieved and reports identified through other methods are listed in Tables S2C and S3. The selection process is presented in a PRISMA flow diagram (Figure 1).

### 2.5. Data Charting

A charting form was piloted on five studies and refined. For each source the following were extracted: first author, year, country, study design, setting, population and sample size, care-continuum stage addressed (recognition, diagnosis, referral, follow-up, physician knowledge), main findings relevant to primary care, and reported gaps or recommendations. The completed charting table is provided in Table S4. The role of primary care in each empirical source was coded as explicit when general practitioners or primary care physicians were a studied population or the stated setting, or when referral from or to primary care was measured; as implied when primary care was not studied directly but its role could be identified from the referral or care pathway described; and as not studied directly when a specialist-setting study reported a care-pathway outcome, such as time to diagnosis, referral or continuity of treatment, without studying primary care itself. Secondary sources in which this variable was not meaningful were coded as not applicable. Full definitions are given in Table S5. Charting was performed by one reviewer, with machine-assisted drafting used only as a supportive aid for some charting text, and verified by a second reviewer against the original sources.

### 2.6. Synthesis

Results were synthesised narratively and tabulated. Sources were first classified by type of evidence (original quantitative, original qualitative, review, guideline) according to the design stated by the publication; a narrative review that also reported descriptive pathway data was classified as secondary, and its descriptive data were used in the synthesis without counting it as an original empirical source. The funding and conflict-of-interest statements published with each included source were also recorded (Table S4). Findings were then mapped onto five stages of the care continuum corresponding to the review questions (evidence map in Table S6). Quantitative findings (prevalence of delay, proportion treated, proportion of physicians adhering to criteria) are reported as published, without pooling, because the populations and definitions were heterogeneous. Deduplication was performed in Python 3.12.3 with pandas 3.0.2 (Table S7); descriptive counts and percentages were generated in Python 3.13.5 using pandas 2.2.3, and figures were produced with matplotlib 3.10.8. No inferential statistical testing was performed.

## 3. Results

### 3.1. Study Selection and Characteristics

The database searches yielded 1327 records (Web of Science 316, PubMed 264, Scopus 747). After removal of 521 duplicates, 806 unique records underwent title and abstract screening; 716 were excluded (702 in the first pass and 14 in the second pass) (the 14 second-pass exclusions comprise 13 of the 38 records re-screened in the second pass and one co-published duplicate removed administratively; of the 41 records identified at the audit, the other two administrative removals, a report already excluded at full text and a source already included, are counted at their original stage, and 25 reports from the second pass proceeded to full-text assessment) and 90 reports were sought for retrieval. Thirteen reports could not be retrieved (Table S2C), leaving 77 reports assessed in full text, of which 31 were excluded (Table S2B) and 46 were included. Fourteen further reports were identified through reference lists, trial registries and targeted web searching; seven were excluded after full-text assessment and seven were included, giving 53 included sources (Figure 1; Table S3). Of these, 31 (58.5%) were original empirical sources and 22 (41.5%) were guidelines or other secondary/prescriptive sources. Original empirical evidence came predominantly from Australia (*n* = 8), the United Kingdom (*n* = 7), the United States (*n* = 7) and Saudi Arabia (*n* = 2), with one source each from France, China, Hungary, Argentina and the United Arab Emirates, one multinational Australia/UK/USA study and one Middle East/Africa regional study (Figure 2). The characteristics of the included sources are summarised in Table 2. Of the 53 sources, 17 declared study or author funding, almost all from public research bodies, professional societies or charities; 19 reported no study-specific or external funding; 13 carried no funding statement; and for four the declaration fields were not accessible. Thirteen sources declared conflicts of interest, mostly editorial-board roles or industry relationships of individual authors, 32 declared none, and eight carried no accessible statement (Table S4).

### 3.2. Recognition and Initial Evaluation in Primary Care

All guideline documents and reviews converge on the same message: POI must be actively considered in every woman under 40 years who presents with oligo- or amenorrhoea of at least four months, infertility or symptoms of oestrogen deficiency, and the initial evaluation (pregnancy test, FSH, oestradiol, thyroid-stimulating hormone, prolactin) is entirely feasible in primary care [1,9,13]. The 2024 guideline simplified the diagnostic criterion to at least four months of disordered cycles plus one FSH concentration above 25 IU/L, with a repeat FSH after four to six weeks or anti-Müllerian hormone measurement reserved for cases of diagnostic uncertainty, and notes explicitly that AMH is still not universally available in primary care [1]. The Australian guideline summary lists early identification, diagnosis, initial chronic disease risk assessment and coordination of referrals as general practitioner responsibilities [9]. Reviews written for family physicians place POI in the routine differential diagnosis of amenorrhoea and set out a first-line laboratory work-up that can be completed in primary care, in a series of American Family Physician reviews updated in 2013, 2019 and 2026 [22,28,35]. A Singaporean practical toolkit developed by a workgroup that included family medicine describes general practitioners as partners in early evaluation and long-term management [33].

Original data on how often this happens in primary care are scarce and indirect. An editorial written by two general practitioners in a primary care journal stated that many GPs have not heard of POI and are not confident diagnosing it and that women are routinely told they are too young to be menopausal or that missed periods need no treatment [26]. In the physician survey from the United Arab Emirates, 92% of respondents recognised more than one symptom suggestive of POI, most often amenorrhoea and hot flushes, yet only 20.4% applied the evidence-based diagnostic criteria, and fewer than half asked about genitourinary or sexual symptoms at the initial assessment [16]. In an Argentinian multicentre registry of 302 women, about half had experienced oligomenorrhoeic cycles for years before diagnosis, and in 27% the aetiological work-up was never completed [46]. In a case series of three adolescents with POI seen in rural general practice in Australia, each had been prescribed the combined oral contraceptive pill for menstrual irregularity before the investigations that led to the diagnosis [40]; the authors’ causal hypothesis regarding vaccination is outside the scope of this review. A survey of 362 Australian clinicians found that GPs order anti-Müllerian hormone testing far less often than specialists (15% vs. 40% monthly) and mostly at patient request, while 22% of all respondents wrongly believed the test can predict premature menopause [55]. Qualitative syntheses of women’s accounts consistently describe menstrual disturbance being attributed to stress, weight or contraception, symptoms being normalised before FSH was measured, and diagnosis as a changeable process that women had to negotiate across providers [32,48]. A recent clinical framework proposes treating the menstrual cycle as a vital sign in primary care, with POI as the sentinel condition that structured assessment must not miss [36].

### 3.3. Diagnostic Delay

Diagnostic delay was the best-documented theme, although no study measured it from the first primary care consultation. In the original NIH survey, more than half of women with spontaneous POI had consulted three or more different clinicians before the diagnosis was made, and in a quarter the interval between onset of menstrual disturbance and diagnosis exceeded five years [10]. Australian women reported a mean delay of up to 1.9 years [11]. In a retrospective case series from an urban safety-net reproductive endocrinology clinic in the United States, 19 of 524 new patients (3.6%) had POI and the median interval from symptom onset to diagnosis was 48 months (interquartile range 23 to 120); 68% already had hypoestrogenic symptoms at referral, 36% of those scanned had low bone mass or osteoporosis, and of the nine women diagnosed before referral only four had started hormone therapy [12]. In a Hungarian university cohort of 125 women with DXA at diagnosis, 43.1% had osteopenia and 10.3% osteoporosis, and time to diagnosis correlated inversely with bone mineral density (r = −0.225, *p* = 0.022) [58]. Among 223 consecutive new referrals to a tertiary POI clinic in the United Kingdom, only 19 of the 79 women with available data (24.1%) presented within 12 months of symptom onset, and reduced bone mineral density was more likely the longer the delay in presentation [20]. A retrospective cohort from Saudi Arabia found that delayed diagnosis, absence of hormone therapy and lack of fertility counselling each independently increased the risk of psychological distress [57]. Qualitative interviews with 24 women with fragile X-associated POI described having to advocate for their own diagnosis and to visit multiple providers before appropriate evaluation, with younger age at onset associated with a longer diagnostic odyssey [54]. In a survey of women recruited from two London multidisciplinary clinics and a national self-help website, only 30% had obtained a diagnosis within six months and 22% had waited up to three years; the authors singled out primary care as the setting where sensitive handling was most often lacking, with women dismissed as too young [38]. In adolescents, two paediatric case series documented a mean interval of 3.8 years between menarche and diagnosis of secondary amenorrhoea-onset POI, incomplete aetiological work-up (FMR1 testing in about 40%, adrenal antibodies in 28 to 29%), and referral for reproductive counselling in under a third [43,47]; a review of adolescent POI attributes the delay to the assumption that irregular menses are common at that age and proposes integrated care coordinated by a primary care clinician [23]. Across sources, the determinants of delay cluster into three groups: low clinician suspicion in young women, intermittent ovarian activity producing fluctuating symptoms and FSH values, and fragmented care across multiple providers.

### 3.4. Referral and Interdisciplinary Care Pathways

Guidelines describe a clear division of labour: primary care initiates the diagnosis, specialist referral is indicated for aetiological work-up (karyotype, FMR1 premutation testing, autoimmune screening), fertility counselling, pubertal induction and complex HT decisions, and primary care resumes responsibility for long-term follow-up [1,9,13]. The recommended referral network includes gynaecology, endocrinology, fertility services, genetic counselling, psychology, physiotherapy and sexual health services [9]. For survivors of childhood and young adult cancer, harmonised surveillance recommendations define who should be monitored for iatrogenic POI and when, a task that in practice often falls to primary care [24].

The empirical evidence on the interface comes from service evaluations in the United Kingdom. In a two-month evaluation of a tertiary (Guy’s Hospital) and a district general (Poole) menopause service, most referrals came from primary care, 30% of new referrals concerned POI, half of referred women had multiple comorbidities, and at the tertiary centre 30% were seen more than 18 weeks after referral; capacity was identified as the main barrier, with non-hormonal options under-used in primary care and alternative follow-up models under-used in clinics [52]. An audit of GP referrals to the same tertiary clinic, which receives about 580 GP referrals a month, found that POI was the second most common referral reason (20%) and that one sixth of referrals could have been managed by alternative routes [56]. An earlier evaluation of the same tertiary clinic (176 new referrals, 2006 to 2010) showed how loosely the interface is timed: 23% of women learned the diagnosis only at their first clinic visit (the source does not distinguish whether the diagnosis had not been made or had not been communicated before referral), 23% were seen within four months of the GP making the diagnosis, 18% between four and twelve months, and 33% more than a year later; a quarter of new appointments were not attended; almost half of women with idiopathic POI initially declined treatment, and women reported GPs advising them to stop HRT because they had been on it for many years, even under the age of 45 [39]. A referral audit at another specialist menopause clinic found POI in only 2 of 50 GP referrals, judged national referral guidance vague and referral patterns haphazard, and developed local pre-referral advice in response [44]. A multidisciplinary review of 103 women diagnosed with POI across six specialty clinics in one teaching hospital found that only 40.6% had a karyotype, 7.4% FMR1 testing and 35.9% a DXA scan (karyotype and FMR1 premutation testing are recommended for all women with non-iatrogenic POI [1]); HRT was discussed with 76%, fertility with 59% and psychological support offered to 25%; investigation and treatment varied significantly between specialties, with reproductive medicine discussing HRT with only 39% of its patients, and the authors proposed a specialist multidisciplinary POI clinic and a proforma [45]. In France, a gynaecological-cardiological pathway that screened 52 women with POI found two or more cardiovascular risk factors in 86.5% before screening and a new risk-factor diagnosis in 43.1% after it, with a letter to the general practitioner as the pathway output; the authors note that no evaluation of such cardio-gynaecological pathways had been published in Europe [37]. Two recent prescriptive sources describe the pathway in more detail. A joint FIGO and IMS position paper names general practitioners among the clinicians who encounter women with POI and proposes an integrated life-course pathway from early identification and risk assessment through hormone therapy to periodic reassessment and longitudinal follow-up, with referral when needed [34], and a Society for Endocrinology guideline developed with primary care input sets out cross-specialty diagnostic steps and referral thresholds for female hypogonadism, including POI [31]. However, no included study evaluated a formal shared-care protocol or care pathway between general practice and gynaecology for POI, and no study measured referral completeness or loss to follow-up at the transition between primary and secondary care. A systematic search for models of care, undertaken to guide development of a POI care model, found that no specific model is recommended for POI and identified 42 components in six themes, with stakeholder engagement central, but it provides a framework without implementation data [30]. The literature on interdisciplinary care for POI is therefore prescriptive rather than empirical.

### 3.5. Long-Term Follow-Up and Hormone Therapy

HT is recommended for all women with POI without contraindications, at doses higher than those used for natural menopause (at least 2 mg oral oestradiol or 100 µg transdermal oestradiol), until at least the average age of menopause, with annual review of cardiovascular risk factors and bone health [1,9]. The evidence on whether this happens indicates substantial implementation gaps. In a multicentre chart review from Saudi Arabia, only 36% of women diagnosed with POI received HT, and uptake remained low even after osteopenia or osteoporosis had been documented [62]. In the United Kingdom tertiary cohort of 223 referred women, 58.7% were taking hormone replacement therapy and 69.5% had undergone bone densitometry [20]. In the physician survey, specialists initiated HT more than twice as often as primary care physicians (65.2% vs. 29.2%), only 55.4% recommended baseline bone densitometry, and one third endorsed at least one intervention without supporting evidence [16]. In the French EMPOIHER cohort of 101 women with spontaneous POI recruited from two university hospitals, 63.4% were receiving menopausal hormone therapy at inclusion, 70% sought additional information from the internet or another practitioner, and psychological support had been offered to only 19.8%; satisfaction with diagnosis disclosure was not associated with adherence to follow-up or hormone therapy [60]. Psychological distress was recognised by 79.3% of surveyed physicians, but fewer than half consistently referred women for psychological support [16]. A qualitative study of 191 women recruited through online support groups described a “care cliff” at midlife, confusion about ongoing treatment and a sense of abandonment by primary care providers, alongside reports of suicidal ideation linked to untreated symptoms [63]. These data concern mainly specialist settings; comparable data on follow-up performed in general practice were not identified.

### 3.6. Knowledge, Attitudes and Practices of Primary Care Physicians

Eight sources measured the knowledge, attitudes or practices of clinicians caring for women with POI, and all included general practitioners or primary care physicians, either as the sole population or alongside specialists. They were conducted in Australia [42,49,51,55], the United States [41,59], the United Arab Emirates [16] and a multinational Middle East and Africa sample [53], with samples ranging from 201 to 2202 respondents. Four domains emerge from these surveys: diagnostic recognition, hormone therapy, awareness of long-term and iatrogenic consequences, and self-reported barriers to care.

Diagnostic recognition was the weakest domain. In the survey of 411 physicians in the United Arab Emirates, only 20.4% applied evidence-based diagnostic criteria for POI, and the gap between recognising symptoms and acting on them according to the guideline was widest in primary care [16]. In Australia, general practitioners ordered anti-Müllerian hormone tests less often than specialists and more often at the patient’s request, while the belief that the test can predict premature menopause, which it cannot, was common across all groups [55]. Practitioners who took part in co-design work in the same country, including general practitioners, themselves identified the need for faster and more empathetic diagnosis [51].

Hormone therapy showed the most consistent difference between primary care and specialist practice. In the United Arab Emirates, HT was initiated by 29.2% of primary care physicians compared with 65.2% of specialists [16]. In a pilot survey at two United States academic centres, 45% of primary care physicians (46/102) always considered HT appropriate for POI, compared with 82% of obstetrician-gynaecologists (47/57) and 86% of endocrinologists (36/42) [59]. In an Australian survey of 745 general practitioners, gynaecologists and endocrinologists, 80% would prescribe HT for vasomotor symptoms in premature menopause, but the combined oral contraceptive pill was the preferred preparation (52%), only 58% would continue treatment to age 51, and the intended duration differed significantly by specialty [42]. In the Middle East and Africa survey, in which family physicians formed the largest group, obstetricians were significantly more likely than the other specialties to treat premature ovarian failure until age 50 [53].

Awareness of long-term and iatrogenic consequences was partial. The largest survey, of 688 Australian general practitioners, measured knowledge of bone health in POI with a 13-item score and found a mean of 9.1, with no difference by practice location [49]. In a nationally representative United States sample, only 15% of 1072 primary care providers, compared with 71% of oncologists, were aware of premature menopause as a late effect of cyclophosphamide [41]. This finding is relevant to iatrogenic POI, because surveillance of cancer survivors for ovarian insufficiency is partly delegated to primary care [24].

Barriers were reported by the clinicians themselves: 41% of the Australian general practitioners described barriers to care for bone health in POI, varying by location [49], and the guideline appraisal that preceded the Australian algorithms found only two of 22 menopause guidelines to be of high quality [29]. On the patient side, a community survey of women of childbearing age in China found constrained knowledge of POI with moderate attitudes and practices [61], and Australian women rated general practitioners as a less expected source of information than gynaecologists or the internet [11]. No European study measuring general practitioners’ knowledge of POI with a structured instrument was identified, and no instrument covering the whole care pathway has been validated for family physicians.

**Figure 3:**
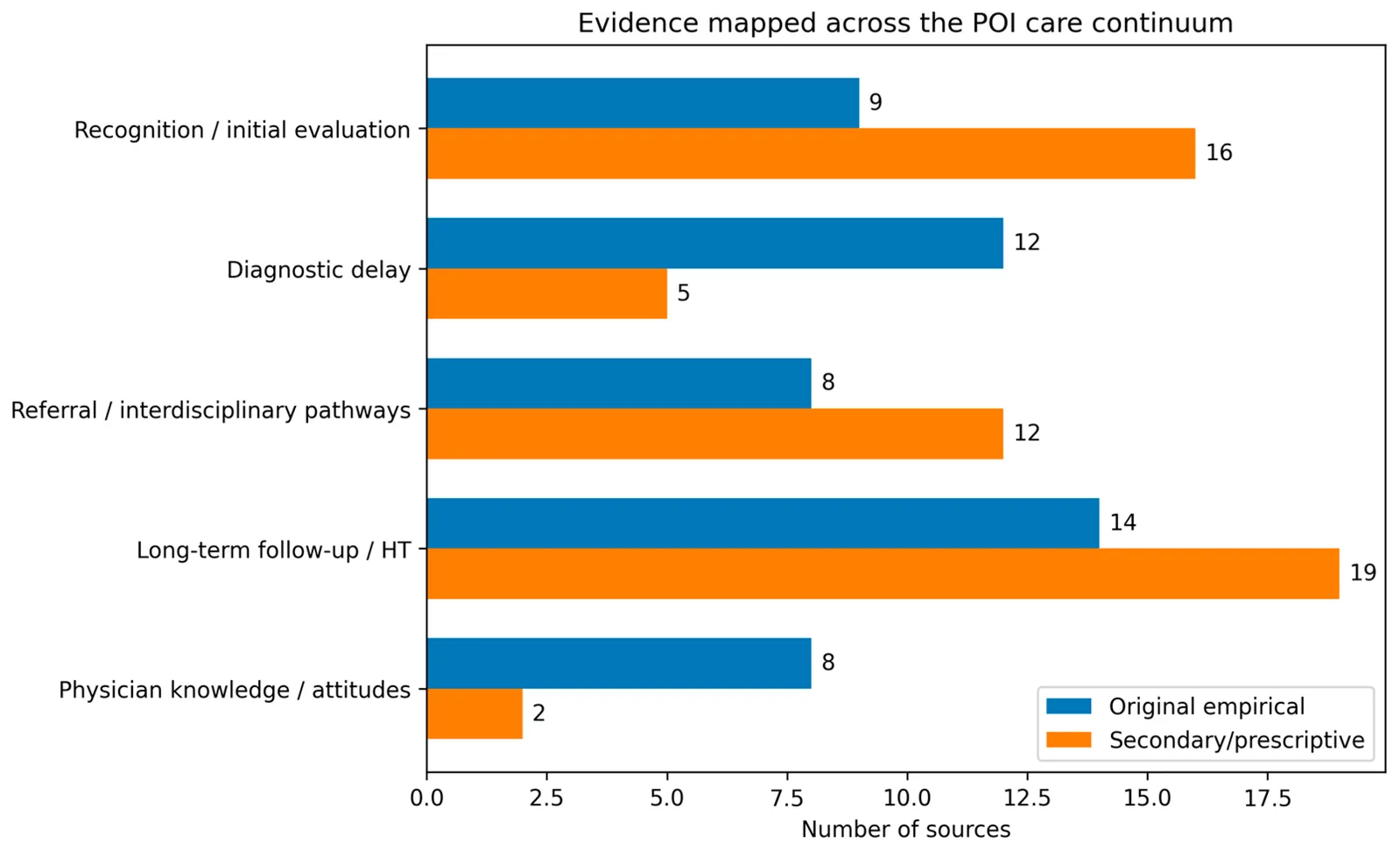
Evidence mapped across the five predefined stages of the POI care continuum. Sources could be assigned to more than one stage; therefore, stage counts are not mutually exclusive.

Across the five predefined care-continuum domains, evidence was unevenly distributed and frequently multi-domain. Original empirical evidence was most frequent for long-term follow-up and hormone therapy (*n* = 14), followed by diagnostic delay (*n* = 12), recognition and initial evaluation (*n* = 9), referral/interdisciplinary pathways (*n* = 8) and physician knowledge and attitudes (*n* = 8). Secondary and prescriptive sources concentrated on recognition and initial evaluation (*n* = 16) and long-term follow-up (*n* = 19). Because individual sources could contribute to more than one domain, counts across domains exceed the total number of included sources (Table S6; Figure 3).

## 4. Discussion

### 4.1. Principal Findings

This review mapped the evidence on primary care involvement across the POI care continuum and found a striking asymmetry. The prescriptive literature is abundant and consistent: the 2024 international guideline, society recommendations and several reviews written for general practitioners consistently assign primary care roles at both ends of the POI care continuum, from recognition at presentation to long-term surveillance [1,9,13,31,33,34]. The empirical literature is thinner and comes mostly from outside primary care. Of 53 included sources, 22 were guidelines or the other secondary/prescriptive literature and 31 reported original empirical data. Within the empirical evidence, the primary care role was explicit in 15 sources and implied in nine; seven specialist-setting studies did not study primary care directly but reported care-pathway outcomes. Despite this breadth, no included study directly observed what happens during a general practice consultation for POI. The original sources are predominantly patient surveys and qualitative studies, specialist service evaluations and audits, and chart reviews and registry analyses, and together they describe a condition that is recognised late, treated inconsistently and followed incompletely: a median of four years from symptoms to diagnosis in one clinic [12], a third of women reaching a tertiary clinic more than a year after the GP made the diagnosis [39], hormone therapy received by only about a third of women with POI in one multicentre chart review [62], and, in two physician surveys, one of them a pilot, lower initiation and endorsement of hormone therapy by primary care physicians than by specialists [16,59]. No study tested an intervention to improve recognition in primary care, and no study evaluated a structured shared-care pathway between general practice and gynaecology.

### 4.2. Why the Gap Persists

We propose four explanatory hypotheses, drawn from the mapped sources rather than tested by them. First, POI has historically been framed as rare and as a specialist condition; the upward revision of prevalence to 3.5% is recent and has not yet changed clinical reflexes [1,2,3]. Second, the presenting symptom is non-specific. Oligo- and amenorrhoea in young women have a broad differential diagnosis and are frequently attributed initially to common physiological, functional or contraceptive causes, and intermittent ovarian activity in POI can produce reassuring intervals and normal hormone values, which lowers suspicion precisely in the group where early treatment matters most [13]. Third, the diagnosis is emotionally charged and the treatment runs against prevailing attitudes to hormone therapy. The physician survey showed that symptom recognition does not translate into guideline-concordant action and that HT is initiated far less often in primary care than by specialists [16], consistent with the broader underuse of HT documented in chart reviews [62]. Women’s accounts add that the diagnosis is often delivered hurriedly and without a plan for what comes next, that general practitioners sometimes advise stopping hormone therapy because of its duration, and that women reaching midlife feel abandoned by services, all of which undermines adherence to a treatment that often needs to continue for many years, generally until the average age of natural menopause [6,10,39,63]. A fourth hypothesis is structural. The service evaluations and audits mapped here describe an interface without a defined owner: referral timing from general practice to specialist care varied from weeks to more than a year [39], investigation and treatment differed by the specialty to which the woman happened to present [45], referrals were often made before guideline-recommended steps had been completed [44,56], and the only interdisciplinary pathway identified, a cardio-gynaecological programme, has as its output a letter to the general practitioner rather than a shared follow-up plan [37]. Where no one owns the transition, delay and discontinuity are predictable outcomes rather than individual failures.

### 4.3. Implications for Family Medicine and Interdisciplinary Care

The mapped evidence supports the development of a testable shared-care model rather than a general appeal to awareness. In primary care, the actionable elements are: a rule that persistent menstrual disturbance (irregular cycles or amenorrhoea for at least four months) in a woman under 40 should prompt consideration of FSH testing; the use of the 2024 criterion (at least four months of disordered cycles plus one FSH concentration above 25 IU/L, with a repeat FSH after four to six weeks if diagnostic uncertainty persists) so that the diagnosis can be made or strongly suspected before referral; a baseline chronic disease risk assessment at diagnosis; and a recall system for annual review of HT adherence, blood pressure, weight and bone health [1,9]. At the interface with gynaecology, the elements are a defined referral content (what the family physician has already done), a defined return content (aetiology, HT regimen, fertility counselling outcome, follow-up plan) and a named owner of long-term surveillance. Several included sources already contain building blocks for this: the structured referral advice developed after a referral audit [44], the multidisciplinary proforma proposed after a review of practice across six specialties [45], the integrated life-course pathway of the FIGO and IMS position paper [34] and the cardiovascular screening programme that reports back to the general practitioner [37]. The international guideline and its primary care summaries provide the clinical content for such a model [1,9,13]; what is missing is its assembly into a single pathway, its implementation in a defined health system and its evaluation against outcomes that matter to women, such as time to diagnosis, HT continuation and bone and cardiovascular surveillance. Table 3 summarises, for each stage of the care continuum, what the mapped evidence shows, where the gap lies and which study design would close it.

### 4.4. Research Gaps

- No validated instrument covering general practitioners’ knowledge and practice across the whole POI care pathway has been published; existing GP surveys address single domains (bone health [49], hormone therapy [42], AMH testing [55]) or mix GPs with specialists [16,59].
- No prospective study has measured the interval from first primary care consultation for menstrual disturbance to confirmed POI diagnosis.
- No study has evaluated a shared-care or care-pathway intervention for POI.
- Data on long-term follow-up come almost exclusively from specialist registries; the trajectory of women discharged back to primary care is unknown.
- Primary-care-specific evidence from Central and Eastern Europe was extremely limited. In Romania, where this review was conducted, no primary care study of POI was identified; the questions that this review could not answer from the literature (how long diagnosis takes, what proportion of women receive and continue HT, and what family physicians know and do) could be addressed by a cross-sectional survey of family physicians and a retrospective cohort of women with POI identified through gynaecology and endocrinology services.

### 4.5. Strengths and Limitations

The review followed JBI methodology and PRISMA-ScR reporting, searched three databases, guideline repositories and reference lists, and used the care continuum as an explicit organising frame, which allowed the asymmetry between the prescriptive and empirical literature to be made visible. Its limitations are those inherent to the design and the field. Restriction to English may have excluded national studies from non-anglophone primary care systems. Only three databases were searched; Embase and CINAHL, which index the additional European, nursing and allied health literature relevant to primary care, were not searched because of lack of institutional access, so some sources may have been missed, although hand-searching of reference lists and guideline repositories partly mitigates this. No included source examined whether the incidence of POI changed after the COVID-19 pandemic, so this question could not be addressed. Quality appraisal was not performed, consistent with scoping review methodology, so included findings carry the limitations of their source designs, in particular small convenience samples, self-reporting and single-centre service evaluations. Most empirical sources came from Australia, the United Kingdom and the United States, so the findings describe anglophone health systems with established general practice; their transferability to systems in which family medicine has a different gatekeeping role is uncertain. Data were charted by one reviewer and verified by a second rather than extracted in duplicate. AI-assisted provisional record classification was used only as a non-decisional organisational aid. During an audit of the screening workbook, 41 records whose abstract-stage closure had been applied inconsistently were identified; after three administrative removals, the remaining 38 were independently re-screened in a blinded second pass under explicit decision rules. Thirteen potentially eligible reports could not be retrieved in full text, most because they were available only by subscription or purchase, which may have resulted in some relevant evidence being missed. Two deviations from the registration occurred. First, inter-reviewer agreement could not be quantified for the complete title and abstract screening set (*n* = 806), or for the initial full-text assessments, because paired reviewer-level decisions were not retained; agreement was therefore quantified for the second-pass screening and for the corrective full-text assessment set comprising the 24 reports advanced from the second pass plus one subsequently reconsidered report. Second, the Scopus language and document-type eligibility criteria were applied during screening rather than as database-interface refinements. In addition, the registered protocol referred to the methodological reviewer as arbiter; following a change in review-team roles, C.A.H. assumed this role. Finally, the field is moving: the 2024 guideline and its primary care summaries are recent, and studies evaluating their uptake may be in progress.

## 5. Conclusions

International guidelines position primary care as a central component of POI care, but it remains poorly studied as a setting. The available evidence indicates delays from symptom onset to diagnosis, gaps in hormone-therapy initiation and persistence, and a lack of formally evaluated shared-care models between family medicine and gynaecology. Closing this gap requires three steps: original data from primary care itself, including the knowledge and practices of family physicians and the real-world trajectory of women from first symptom to long-term follow-up; a shared-care pathway that specifies what general practice does before referral, what specialist care returns, and who owns long-term surveillance; and evaluation of that pathway against time to diagnosis, hormone therapy continuation and bone and cardiovascular surveillance. The present mapping provides the rationale, the organising frame and the stage-specific research agenda for such studies.

## Figures and Tables

**Table 1:** Eligibility criteria according to the Population, Concept, Context (PCC) framework. HT, hormone therapy; POI, premature ovarian insufficiency.

Element	Inclusion	Exclusion
Population	Women with POI (spontaneous or iatrogenic), diagnosed before 40 years, or physicians caring for them	Early menopause (40–45 years) only; Turner syndrome cohorts without a primary care dimension
Concept	Recognition, initial evaluation, diagnostic delay, referral, interdisciplinary pathways, long-term follow-up, HT adherence, physician knowledge or attitudes	Pathophysiology, genetics, fertility treatment or laboratory diagnostics without reference to the care pathway
Context	Primary care, general practice, family medicine, or an identifiable interface with primary care through referral, diagnostic delay, treatment continuity or longitudinal follow-up	Tertiary or fertility-centre studies with no primary-care role and no identifiable referral or care-pathway outcome
Sources	Original studies of any design (quantitative, qualitative, mixed), guideline documents, and narrative or systematic reviews with POI-specific care-pathway relevance	Conference abstracts without full text, letters, case reports of a single patient
Language and date	English-language full text; database inception to 4 October 2026	Full text not available in English

**Table 2:** Characteristics of the 53 included sources, ordered by evidence type (guidelines and secondary sources first) and year. The main finding of each source relevant to primary care is given in Table S4.

First Author, Year	Country	Design	Care-Continuum Stage	Primary Care Role
Davies 2012 [21]	United Kingdom	Expert review (clinical question)	Recognition; diagnostic delay; referral/pathways; follow-up/HT	Implied
Klein 2013 [22]	USA	Review for family physicians (amenorrhoea)	Recognition; follow-up/HT	Explicit
Gordon 2015 [23]	USA	Narrative review (adolescents)	Recognition; diagnostic delay; referral/pathways	Explicit
van Dorp 2016 [24]	International	Harmonised surveillance guideline	Referral/pathways; follow-up/HT	Implied
Lukman 2017 [14]	United Kingdom	Textbook chapter for primary care	Recognition; follow-up/HT	Explicit
Nguyen 2017 [15]	Australia	Review for GPs with index case	Recognition; referral/pathways; follow-up/HT	Explicit
Machura 2018 [25]	Poland	Narrative review	Follow-up/HT	Not applicable
Newson 2018 [26]	United Kingdom	Editorial by GPs	Recognition; diagnostic delay; follow-up/HT; knowledge/attitudes	Explicit
Kiriakova 2019 [27]	Australia	Guideline appraisal (AGREE II), 16 guidelines	Follow-up/HT	Not applicable
Klein 2019 [28]	USA	Review for family physicians (amenorrhoea)	Recognition; follow-up/HT	Explicit
Yeganeh 2019 [29]	Australia	Guideline appraisal, 22 guidelines	Recognition; follow-up/HT	Not applicable
Jones 2020 (SRM) [30]	Australia	Systematic search and narrative review	Referral/pathways	Not applicable
Lambrinoudaki 2021 [13]	International (EMAS/IMS)	Expert review for primary care	Recognition; diagnostic delay; referral/pathways; follow-up/HT; knowledge/attitudes	Explicit
Jayasena 2024 [31]	United Kingdom	Society for Endocrinology guideline (female hypogonadism)	Recognition; referral/pathways; follow-up/HT	Explicit
Panay 2024 [1]	International	Evidence-based guideline	Recognition; referral/pathways; follow-up/HT	Explicit
Hammond 2025 [32]	United Kingdom	Thematic synthesis, 10 qualitative studies	Diagnostic delay; follow-up/HT	Implied
Sugianto 2025 [33]	Singapore	Practical toolkit (workgroup including family medicine)	Recognition; referral/pathways; follow-up/HT	Explicit
Benedetto 2026 [34]	International	Joint FIGO/IMS position paper	Recognition; referral/pathways; follow-up/HT	Implied
Klein 2026 [35]	USA	Review for family physicians (amenorrhoea)	Recognition; follow-up/HT	Explicit
Nelson 2026 [36]	USA	Clinical framework	Recognition	Explicit
Vincent 2026 [9]	Australia	Guideline summary for general practitioners	Recognition; referral/pathways; follow-up/HT	Explicit
Cavadias 2026 [37]	France	Narrative review reporting descriptive data from a cardio-gynaecological pathway (52 women with POI)	Referral/pathways; follow-up/HT	Implied
Alzubaidi 2002 [10]	USA	Patient survey (*n* = 50)	Diagnostic delay	Implied
Deeks 2011 [11]	Australia	Patient survey	Diagnostic delay	Implied
Singer 2011 [38]	United Kingdom	Survey, two clinics and self-help website	Diagnostic delay; follow-up/HT	Explicit
Cartwright 2012 [39]	United Kingdom	Service evaluation, tertiary POF clinic (176 new referrals)	Referral/pathways; follow-up/HT	Explicit
Little 2014 [40]	Australia	Case series, 3 adolescents seen in general practice	Recognition	Explicit
Mittal 2014 [20]	United Kingdom	Retrospective review, 223 consecutive referrals to a tertiary POI clinic	Diagnostic delay; referral/pathways; follow-up/HT	Not studied directly
Nekhlyudov 2014 [41]	USA	National survey, 1130 oncologists and 1072 PCPs	Knowledge/attitudes	Explicit
Yeganeh 2017 [42]	Australia	Online survey, 745 HPs	Knowledge/attitudes	Explicit
Kanj 2018 [43]	USA	Chart review, 50 adolescents and young adults	Recognition; follow-up/HT	Not studied directly
Neill 2018 [44]	United Kingdom	Referral audit, 50 GP letters	Referral/pathways	Explicit
Richardson 2018 [45]	United Kingdom	Multidisciplinary review of practice, 103 women	Recognition; referral/pathways; follow-up/HT	Not studied directly
Demayo 2019 [46]	Argentina	Multicentre registry, 302 women	Recognition; diagnostic delay	Not studied directly
Hoyos-Martinez 2019 [47]	USA	Case series, 7 idiopathic adolescents	Recognition; diagnostic delay; referral/pathways	Implied
Johnston-Ataata 2020 [48]	Australia	Narrative interviews, 30 women	Diagnostic delay	Implied
Jones 2020 (Arch Osteoporos) [49]	Australia	Online surveys, 688 GPs and 316 women	Follow-up/HT; knowledge/attitudes	Explicit
Yeganeh 2020 (Menopause) [50]	Australia	Co-design, 263 women, pilot	Follow-up/HT	Implied
Yeganeh 2020 (SRM) [51]	Australia	Mixed-methods co-design	Recognition; knowledge/attitudes	Explicit
Manley 2021 [52]	United Kingdom	Service evaluation, tertiary and district clinics	Referral/pathways	Explicit
Minis 2022 [12]	USA	Case series, safety-net clinic, 19 of 524 patients	Diagnostic delay; referral/pathways; follow-up/HT	Implied
Beshyah 2023 [53]	Middle East and Africa	Survey, 254 physicians (36% family medicine)	Knowledge/attitudes	Explicit
Poteet 2023 [54]	USA	Qualitative interviews, 24 women with FXPOI	Diagnostic delay	Implied
Copp 2024 [55]	Australia	Nationwide survey, 362 clinicians	Recognition; knowledge/attitudes	Explicit
Tedajo-Tsambou 2024 [56]	United Kingdom	Audit, 50 GP referrals to tertiary clinic	Referral/pathways	Explicit
Bakhsh 2025 [57]	Saudi Arabia	Retrospective cohort	Diagnostic delay; follow-up/HT	Not studied directly
Csehely 2025 [58]	Hungary	Retrospective cohort, 125 women	Diagnostic delay	Not studied directly
Jeevananthan 2025 [59]	USA	Provider survey, 201 (102 PCPs)	Follow-up/HT; knowledge/attitudes	Explicit
Aubin 2026 [60]	France	Multicentre cohort, 101 women with spontaneous POI (EMPOIHER)	Follow-up/HT	Implied
Langhe 2026 [16]	United Arab Emirates	Physician survey, 411	Recognition; follow-up/HT; knowledge/attitudes	Explicit
Liu 2026 [61]	China	Community KAP survey	Recognition	Implied
Malick 2026 [62]	Saudi Arabia	Chart review, three tertiary hospitals	Follow-up/HT	Not studied directly
Pisana 2026 [63]	Australia, UK, USA	Online qualitative, 191 women	Diagnostic delay; follow-up/HT	Explicit

Abbreviations: AGREE II, Appraisal of Guidelines for Research and Evaluation II; FXPOI, fragile X-associated primary ovarian insufficiency; GP, general practitioner; HP, healthcare professional; HT, hormone therapy; KAP, knowledge, attitudes and practices; PCP, primary care physician; POF, premature ovarian failure; POI, premature ovarian insufficiency; SRM, Seminars in Reproductive Medicine.

**Table 3:** Evidence, gaps and research priorities by stage of the POI care continuum. GP, general practitioner; HT, hormone therapy; AMH, anti-Müllerian hormone; KAP, knowledge, attitudes and practices.

Stage	What the Mapped Evidence Shows	Main Gap	Priority Study Design
Recognition and initial evaluation	Guidance for family physicians is abundant and consistent [1,9,13,14,15,22,28,35]; only one empirical source was generated in general practice itself, a series of three adolescents prescribed combined oral contraception before investigation [40]; 20.4% of physicians applied evidence-based diagnostic criteria in one survey [16]	No measurement of recognition at the first primary care consultation	Prospective cohort of women presenting to general practice with menstrual disturbance, with FSH testing and diagnosis as outcomes
Diagnostic delay	Delay of one to several years is consistently reported from patient surveys, qualitative studies and specialist cohorts [10,11,12,38,48,54,58,63]; longer delay is associated with lower bone density [20,58] and greater psychological distress [57]	Delay is measured from symptom onset or from referral, never from the first primary care contact	Retrospective cohort linking primary care and specialist records, with interval from first consultation to diagnosis as the primary outcome
Referral and interdisciplinary pathways	POI accounts for 20 to 30% of new referrals to specialist menopause clinics, mostly from primary care [52,56]; referral timing and content vary widely [39,44]; investigation differs by presenting specialty [45]; one cardio-gynaecological pathway exists but is not evaluated [37]	No shared-care protocol between general practice and gynaecology has been implemented or evaluated	Implementation study of a structured referral and return pathway with a named owner of long-term surveillance
Long-term follow-up and hormone therapy	HT initiation was lower among primary care physicians than specialists in one survey [16], and another found substantially lower endorsement of HT as appropriate among primary care physicians [59]; about one third of women in one multicentre chart review received HT [62]; a narrative review reports that a substantial proportion discontinue within the first year [25]; women describe a care cliff at midlife [63]	The trajectory of women discharged from specialist care to general practice is unknown	Registry or cohort study of HT persistence and bone and cardiovascular surveillance after discharge to primary care
Physician knowledge and attitudes	Surveys of general practitioners address single domains, such as bone health [49], hormone therapy [42] or AMH testing [55], or combine GPs with specialists [16,53,59]; no European study of family physicians was identified	No validated instrument covering GP knowledge and practice across the whole POI pathway	Development and validation of a KAP instrument for family physicians, followed by a national survey

## Data Availability

The retained review materials, including the deduplicated record set (*n* = 806), retained screening and full-text reconciliation records, final verified data-charting workbook, synthesis tables, Supplementary Material and deduplication script, will be made openly available upon publication of the article, under a CC BY 4.0 licence, in the OSF project linked to the protocol registration (https://osf.io/erf27 (accessed on 6 October 2026); DOI: 10.17605/OSF.IO/ERF27). A README will describe the file structure, variables, coding conventions and known limitations. Full texts of included publications will not be shared because of copyright and licensing restrictions.

## Supplementary Materials

The following supporting information can be downloaded at https://www.mdpi.com/article/10.3390/healthcare14213795/s1: Table S1: Complete electronic search strategies; Table S2A: Records excluded at the second-pass stage (*n* = 14); Table S2B: Reports excluded after full-text assessment (database searches; *n* = 31); Table S2C: Reports sought but not retrieved (*n* = 13); Table S3: Reports identified through other methods (*n* = 14); Table S4: Data-charting table for the 53 included sources; Table S5: Coding framework, operational definitions and screening decision rules; Table S6: Evidence map: contribution of each included source to the five care-continuum stages; Table S7: Deduplication procedure. File S1: PRISMA-ScR checklist.

## Abbreviations

The following abbreviations are used in this manuscript: AMH, anti-Müllerian hormone; ASRM, American Society for Reproductive Medicine; BMD, bone mineral density; COCP, combined oral contraceptive pill; CRE-WHiRL, Centre for Research Excellence in Women’s Health in Reproductive Life; DXA, dual-energy X-ray absorptiometry; ESHRE, European Society of Human Reproduction and Embryology; FIGO, International Federation of Gynecology and Obstetrics; FSH, follicle-stimulating hormone; FXPOI, fragile X-associated primary ovarian insufficiency; GP, general practitioner; HT, hormone therapy; HRT, hormone replacement therapy; IMS, International Menopause Society; JBI, Joanna Briggs Institute; KAP, knowledge, attitudes and practices; OSF, Open Science Framework; PCC, population, concept, context; PCP, primary care physician; POI, premature ovarian insufficiency; PRISMA-ScR, Preferred Reporting Items for Systematic reviews and Meta-Analyses extension for Scoping Reviews.
