# Supplementary material for: Premature Ovarian Insufficiency in Primary Care: A Scoping Review of Recognition, Diagnostic Delay and Interdisciplinary Care Pathways
Source: Healthcare (Basel). 2026 Nov 6;14(21):3795. doi: 10.3390/healthcare14213795 (PMC13762931; doi:10.3390/healthcare14213795)
Supplement: Supplementary file 1 [file healthcare-14-03795-s001.zip › PRISMA-ScR_checklist.pdf]

PRISMA-ScR checklist

Premature Ovarian Insufficiency in Primary Care: A Scoping Review of Recognition, Diagnostic Delay and Interdisciplinary Care Pathways. Checklist items from Tricco et al., Ann. Intern. Med. 2018, 169, 467–473.

Page references refer to the revised manuscript with tracked changes displayed (25 pages).

Item	Section/topic	Checklist item (abridged)	Reported on page
1	Title	Identify the report as a scoping review	Title, p. 1
2	Structured summary	Background, objectives, eligibility, sources, charting, results, conclusions	Abstract, pp. 1–2
3	Rationale	Rationale in the context of what is known	~~Section 1, pp. 2–3~~Section 1, pp. 2–3
4	Objectives	Questions and objectives (population, concept, context)	~~Section 1, p. 3; Section 2.2, p. 3~~Section 1, p. 3; Section 2.2, pp. 3–4
5	Protocol and registration	Protocol, registration and deviations	~~Section 2.1, p. 3; deviations in Section 4.5, p. 17~~Section 2.1, p. 3; deviations in Section 4.5, pp. 19–20
6	Eligibility criteria	Characteristics used as criteria, with rationale	~~Section 2.2, p. 3; Table S5~~Section 2.2, pp. 3–4; Table S5
7	Information sources	Databases, other sources, date of last search	~~Section 2.3, p. 4; Table S1~~Section 2.3, pp. 4–5; Table S1
8	Search	Full electronic search strategy	~~Section 2.3, p. 4; Table S1~~Section 2.3, pp. 4–5; Table S1
9	Selection of sources of evidence	Screening and eligibility process	~~Section 2.4, pp. 4–5; Tables S2A–S2C~~Section 2.4, p. 5; Tables S2A–S2C
10	Data charting process	Methods of charting and verification	~~Section 2.5, p. 5~~Section 2.5, pp. 5–6
11	Data items	Variables for which data were sought	~~Section 2.5, p. 5; Table S5~~Section 2.5, pp. 5–6; Table S5
12	Critical appraisal (optional)	Rationale and methods if done	~~Section 2.1, p. 3 (not performed)~~Section 2.1, p. 3 (not performed)
13	Synthesis of results	Methods of handling and summarising data	~~Section 2.6, p. 5~~Section 2.6, p. 6
14	Selection of sources of evidence	Numbers screened, assessed and included, with reasons; flow diagram	~~Section 3.1, p. 5; Figure 1, p. 6; Tables S2A–S2C, S3~~Section 3.1, pp. 6–7; Figure 1, p. 7; Tables S2A–S2C, S3
15	Characteristics of sources	Characteristics for which data were charted	~~Section 3.1, p. 5; Table 1, pp. 6–8; Table S4~~Section 3.1, pp. 6–7; Table 1, pp. 7–10; Table S4
16	Critical appraisal within sources (optional)	Data on critical appraisal	Not applicable
17	Results of individual sources	Relevant data charted for each source	~~Table 1, pp. 6–8; Table S4~~Table 1, pp. 7–10; Table S4

Item	Section/topic	Checklist item (abridged)	Reported on page
18	Synthesis of results	Results in relation to the review questions	~~Sections 3.2–3.6, pp. 9–14; Figures 2–4, pp. 13–14; Table S6~~Sections 3.2–3.6, pp. 10–14; Figures 2–3, pp. 15–16; Table S6
19	Summary of evidence	Main results and relevance	~~Section 4.1, p. 14; Table 2, p. 16~~Section 4.1, p. 16; Table 2, p. 19
20	Limitations	Limitations of the review process	~~Section 4.5, p. 17~~Section 4.5, pp. 19–20
21	Conclusions	Interpretation and implications	~~Section 5, p. 18~~Section 5, p. 20
22	Funding	Sources of funding for the included sources and for the review	~~Section 3.1, p. 5; Table S4; Funding statement, p. 18~~Section 3.1, pp. 6–7; Table S4; Funding statement, p. 21
