# Supplementary material for: Premature Ovarian Insufficiency in Primary Care: A Scoping Review of Recognition, Diagnostic Delay and Interdisciplinary Care Pathways
Source: Healthcare (Basel). 2026 Nov 6;14(21):3795. doi: 10.3390/healthcare14213795 (PMC13762931; doi:10.3390/healthcare14213795)
Supplement: Supplementary file 1 [file healthcare-14-03795-s001.zip › healthcare-4641852-supplementary file.pdf]

Supplementary Material

Premature Ovarian Insufficiency in Primary Care: A Scoping Review of Recognition, Diagnostic Delay and Interdisciplinary Care Pathways

Iulia Silcău, Ciprian-Nicușor Solomon, Laura Maghiar, Ioana Adelina Faur, Irina Daniela Henegariu, Luana Ghilea (Seles), Cristian Sava, Alin Bodog, Teodor Andrei Maghiar and Carmen Anca Huniadi

Table S1. Complete electronic search strategies. All searches were run from database inception to 4 October 2026.

Database	Interface and date	Search string	Filters	Records retrieved
PubMed/MEDLINE	NCBI PubMed; 4 October 2026	`("Primary Ovarian Insufficiency"[Mesh] OR "premature ovarian insufficiency"[tiab] OR "primary ovarian insufficiency"[tiab] OR "premature ovarian failure"[tiab] OR "premature menopause"[tiab] OR "early menopause"[tiab] OR "hypergonadotropic amenorrh*"[tiab]) AND ("Primary Health Care"[Mesh] OR "General Practice"[Mesh] OR "General Practitioners"[Mesh] OR "Physicians, Family"[Mesh] OR "Physicians, Primary Care"[Mesh] OR "Family Practice"[Mesh] OR "Referral and Consultation"[Mesh] OR "Delayed Diagnosis"[Mesh] OR "primary care"[tiab] OR "general practi*"[tiab] OR "family physician*"[tiab] OR "family doctor*"[tiab] OR "family medicine"[tiab] OR "diagnostic delay"[tiab] OR "delayed diagnosis"[tiab] OR "time to diagnosis"[tiab] OR referral[tiab] OR "care pathway*"[tiab] OR "shared care"[tiab] OR interdisciplinary[tiab] OR multidisciplinary[tiab] OR "knowledge, attitudes"[tiab] OR "physician survey"[tiab]) AND humans[filter] AND english[lang]`	Humans; English (within the search string)	264
Scopus	Elsevier Scopus, Advanced search, institutional access; 4 October 2026	`TITLE-ABS-KEY("premature ovarian insufficiency" OR "primary ovarian insufficiency" OR "premature ovarian failure" OR "premature menopause" OR "early menopause" OR "hypergonadotropic amenorrh*") AND TITLE-ABS-KEY("primary care" OR "primary health care" OR "general practi*" OR "family physician*" OR "family doctor*" OR "family medicine" OR "diagnostic delay" OR "delayed diagnosis" OR "time to diagnosis" OR referral OR "care pathway*" OR "shared care" OR interdisciplinary OR multidisciplinary OR "knowledge, attitudes" OR "physician survey")`	English; Article or Review (applied during screening)	747 (614 met the language and document-type criteria)
Web of Science Core Collection	Clarivate Web of Science, Advanced search, institutional access; 4 October 2026	`TS=("premature ovarian insufficiency" OR "primary ovarian insufficiency" OR "premature ovarian failure" OR "premature menopause" OR "early menopause" OR "hypergonadotropic amenorrh*") AND TS=("primary care" OR "primary health care" OR "general practi*" OR "family physician*" OR "family doctor*" OR "family medicine" OR "diagnostic delay" OR "delayed diagnosis" OR "time to diagnosis" OR referral OR "care pathway*" OR "shared care" OR interdisciplinary OR multidisciplinary OR "knowledge, attitudes" OR "physician survey")`	English; Article, Review Article	316

Note. The same free-text terms were used in all three databases; MeSH terms were used in PubMed only. All 747 Scopus records entered deduplication; the language and document-type criteria were applied during screening rather than in the Scopus interface, which differs from the registered protocol.

Table S2A. Records excluded at the second-pass stage (*n* = 14): 13 records excluded after independent re-screening of the 38 records and one co-published duplicate (No. 2) removed administratively.

No.	Record	Reason for exclusion
2	Benedetto et al., 2026. Hormone therapy (HT) in women with premature ovarian insufficiency or early menopause: Time to think of a new paradigm for healthy aging. A joint FIGO and IMS position paper	Co-published version of record No. 1 (same position paper in a second journal); removed as a duplicate publication (R3).
7	Yongue et al., 2026: Menopause following cancer treatment-induced menopause: unique considerations	Cancer treatment-induced menopause across ages; the abstract does not provide a distinct POI-specific primary-care, referral, or follow-up pathway.
9	Anthon et al., 2024: Menstrual Disorders in Adolescence: Diagnostic and Therapeutic Challenges	General adolescent menstrual-disorder review; no distinct POI-specific primary-care or care-pathway content is evident from the abstract.
11	Naseem et al., 2023: Management of congenital hypogonadotropic hypogonadism in females	Congenital hypogonadotropic hypogonadism rather than POI.

No.	Record	Reason for exclusion
12	Gaspari et al., 2023: Primary Amenorrhea in Adolescents: Approach to Diagnosis and Management	General primary-amenorrhoea review; no distinct POI-specific care-pathway content is evident from the abstract.
13	Wesevich et al., 2020: Recent advances in understanding primary ovarian insufficiency	Clinical/mechanistic update focused predominantly on pathophysiology and genetics, without an identifiable primary-care or care-pathway dimension.
22	Armon et al., 2023: BRCA carriers after risk-reducing bilateral salpingo-oophorectomy	Risk-reducing bilateral salpingo-oophorectomy/surgical-menopause population up to age 50; no separable POI population is evident.
23	Iñesta et al., 2023: Knowledge, attitudes and experiences of menopause among “early” and “on-time” groups	Early-menopause versus on-time-menopause study; no separable POI (<40 years) population is evident from the abstract.
27	Currie et al., 2021: Vision for menopause care in the UK	General menopause-care vision; no distinct POI-specific recognition, referral or follow-up content in the abstract (R2).
28	Absatarova et al., 2025: The role of an interdisciplinary approach to the treatment of patients with reduced ovarian reserve	Two clinical cases centred on fertility treatment and mood disorders; no primary-care, referral, or continuity pathway relevant to the PCC.
34	Armeni et al., 2016: Maintaining postreproductive health: A care pathway from EMAS	General postreproductive and menopause care pathway; no distinct POI-specific content in the abstract (R2).
35	Kozica-Olenski et al., 2023: The impact of COVID-19 on a specialised menopause clinic	Specialist menopause-clinic service-delivery study; no separable POI population or primary-care POI pathway in the abstract.
38	Schmidt et al., 2010: Late effects in adult survivors of childhood cancer: considerations for the general practitioner	General childhood-cancer survivorship review; the abstract contains no distinct POI-specific recognition, referral or follow-up content (R2).
41	Boothroyd, 2002: Amenorrhoea: A guide for GPs	General amenorrhoea guide for GPs; the abstract lacks distinct POI-specific recognition, referral or follow-up content (R2).

Note. Forty-one records initially closed at the abstract stage were re-screened in a second pass under the decision rules in Table S5. Three were removed administratively before re-screening: No. 2, a co-published duplicate (listed above); No. 14, a report already excluded after full-text assessment; and No. 20, a source already included. Of the remaining 38 records, the two reviewers (I.S., L.M.), each blinded to the other's decisions, agreed on 35 (92.1%; Cohen's κ = 0.84). The three disagreements were resolved according to the registered conservative rule whereby any record retained by either reviewer proceeded to full-text assessment. Twenty-four records proceeded to full text and 14 were initially excluded; one of the 14 (No. 39, Mittal 2014) was subsequently reconsidered for consistency with the 'not studied directly' rule and assessed in full text, leaving the 13 second-pass exclusions listed above.

Table S2B. Reports excluded after full-text assessment (database searches; *n* = 31).

Report	Title	Reason category	Reason for exclusion
Reilly, 2001	Premature Menopause: A Multidisciplinary Approach Whurr	Ineligible source type	Source/context not eligible: short review/commentary of a book rather than a substantive original study, guideline or primary-care review with chartable POI pathway data.
Pines et al., 2008	HRT in the early menopause: Scientific evidence and common perceptions	Population or concept not POI-specific	R5: 'early menopause' in this position paper refers to early postmenopausal treatment timing, not POI or an eligible early-menopause cohort.
Cooper et al., 2011	The time is now for a new approach to primary ovarian insufficiency	No primary care role or identifiable care pathway	PCC context not met: conceptual multidisciplinary/registry proposal without a distinct primary-care role or primary-care pathway outcome.
Seifer, 2011	Strategic planning is long overdue and could mitigate long-term complications that result from delayed diagnosis of primary ovarian insufficiency	No primary care role or identifiable care pathway	Editorial commentary on research strategy; no primary care content

Report	Title	Reason category	Reason for exclusion
Arora et al., 2011	Diagnosis and management of premature ovarian failure	No primary care role or identifiable care pathway	Review for obstetricians and gynaecologists; no primary care content
Sayakhot et al., 2013	Differences in clinician understanding and management of early menopause after breast cancer	Population or concept not POI-specific	The participants were 176 clinicians, including 35 GPs. The clinical scenario and practice questions concerned early menopause after breast cancer in women <45 years; a POI <40 subgroup was not separable.
Mittal et al., 2013	A cross-sectional national survey assessing the clinical attitudes to the management of premature ovarian insufficiency	Specialist-only clinician survey (S1)	S1: survey of British Menopause Society members, predominantly specialist clinicians, without a primary-care subgroup or referral/shared-care outcome.
Pitkin, 2013	Should HRT be duration limited?	No primary care role or identifiable care pathway	Personal view on HRT duration; mentions GPs limiting HRT to 5 years but no POI pathway data
American College of Obstetricians and Gynecologists, 2014	Primary Ovarian Insufficiency in Adolescents and Young Women	No primary care role or identifiable care pathway	PCC context not met: ACOG specialist committee opinion, not a primary-care-directed source and without an explicit primary-care pathway.
Baber et al., 2014	Primary Ovarian Insufficiency	No primary care role or identifiable care pathway	General review; multidisciplinary approach recommended but no primary care content
Lindh-Åstrand et al., 2015	Hormone therapy might be underutilized in women with early menopause	Population or concept not POI-specific	Population consisted exclusively of women aged 40–44 years with assumed early menopause; this matches the registered early-menopause-only exclusion.
Sarri et al., 2015	Diagnosis and management of menopause: Summary of NICE guidance	No primary care role or identifiable care pathway	PCC context not met: general NICE menopause summary rather than a primary-care/POI-focused source; POI recommendations are not sufficient to establish an eligible care setting.
Jamieson, 2015	Disorders of Menstruation in Adolescent Girls	Population or concept not POI-specific	General adolescent menstrual disorders; POI mentioned only in differential
Webber et al., 2016	ESHRE Guideline: management of women with premature ovarian insufficiency	Duplicate, co-publication or superseded version (R3)	Superseded by the 2024 guideline (included), which replaces it; counted once as the current version.
Heida et al., 2016	Cardiovascular risk management after reproductive and pregnancy-related disorders: A Dutch multidisciplinary evidence-based guideline	No primary care role or identifiable care pathway	POI addressed, but no explicit primary care role or identifiable POI-specific referral or follow-up pathway (arbitration).
Tønnes Pedersen et al., 2018	Transition in Pediatric and Adolescent Hypogonadal Girls: Gynecological Aspects, Estrogen Replacement Therapy, and Contraception.	No primary care role or identifiable care pathway	Transition of hypogonadal girls; hormonal management, no primary care content

Report	Title	Reason category	Reason for exclusion
Jarcevic et al., 2018	Survey of Canadian PGY-4 and PGY-5 Obstetrics and Gynaecology Residents on Knowledge of and Exposure to Premature Ovarian Insufficiency	Specialist-only clinician survey (S1)	Participants were Canadian PGY-4/PGY-5 obstetrics and gynaecology residents only. No primary-care subgroup or pathway outcome.
Marsden et al., 2019	British Menopause Society consensus statement on the management of estrogen deficiency symptoms, arthralgia and menopause diagnosis in women treated for early breast cancer	Population or concept not POI-specific	R2/PCC: breast-cancer treatment-induced menopause population is not restricted to POI <40 and a separable POI subgroup is not available.
Berga et al., 2019	Barriers to the Care of Menopausal Women	Population or concept not POI-specific	Menopause care in general; POI is not a distinct, separable population or care pathway (R2; arbitration).
Siyam et al., 2019	Determinants of hormone therapy uptake and decision-making after bilateral oophorectomy (BO): A narrative review	No primary care role or identifiable care pathway	Narrative review on HT after bilateral oophorectomy; surgical menopause decision-making, no POI pathway content
Wang et al., 2020	Knowledge of iatrogenic premature ovarian insufficiency among Chinese obstetricians and gynaecologists: a national questionnaire survey	Specialist-only clinician survey (S1)	National survey of obstetricians and gynaecologists. Although hospital level was analysed and 'primary care hospitals' are mentioned, the sampled clinicians were not primary-care physicians and no referral/shared-care pathway was studied.
Fruzzetti et al., 2020	Tailored hormonal approach in women with premature ovarian insufficiency.	No primary care role or identifiable care pathway	Review of hormonal therapy; no primary care or care-pathway content
Dempsey, 2020	Evidence-based management of premature ovarian insufficienc	Ineligible source type	Single-page item in a journal special issue; only abstract-length text was available, so it was treated as a conference abstract without full text (registered source-type criterion).
Lambrinoudaki et al., 2021	Premature ovarian insufficiency: A toolkit for the primary care physician*	Duplicate, co-publication or superseded version (R3)	Co-publication of an included report (same toolkit in Climacteric and Maturitas); counted once (R3).
Alipoor et al., 2024	The Necessity of Using the Registry Systems for Primary Ovarian Insufficiency	No primary care role or identifiable care pathway	PCC concept/context not met: registry-systems chapter focused on data infrastructure rather than a primary-care recognition, referral or follow-up pathway.
Silva et al., 2024	Premature ovarian insufficiency: knowledge, attitudes, and quality of care offered by gynecologists	Specialist-only clinician survey (S1)	S1: specialist-only empirical clinician survey: participants were gynaecologists, with no separable GP/family-medicine subgroup or shared-care outcome.
Cipres et al., 2024	Primary Ovarian Insufficiency, Bone Health, and Other Outcomes in Adolescents.	No primary care role or identifiable care pathway	Context criterion not met: an identifiable POI care pathway is described, but no primary care role is described or identifiable from it (arbitration; applied consistently with FT05, FT10, FT12 and FT17).

Report	Title	Reason category	Reason for exclusion
Singleton et al., 2025	Women’s healthcare providers’ knowledge and practices surrounding fragile-X associated primary ovarian insufficiency (FXPOI)	Specialist-only clinician survey (S1)	Women's-health provider survey: general OBGYN, MFM, REI and related women's-health specialties; no primary-care/family-medicine subgroup and no referral/shared-care outcome.
Peery et al., 2026	Improving women's healthcare providers' knowledge about fragile X-associated primary ovarian insufficiency through a novel educational tool	Specialist-only clinician survey (S1)	Educational intervention in women's-health specialties (predominantly OBGYN, with REI/MFM and related providers); no primary-care subgroup and no referral/shared-care outcome.
Capozzi et al., 2026	Expert opinion by the Italian Society of Gynecology of the Third Age (SIGiTE) and the Italian Society of Menopause (SIM) on diagnosis and treatment of premature ovarian insufficiency	No primary care role or identifiable care pathway	PCC context not met: specialist society expert opinion without a defined primary-care audience or explicit primary-care role.
Ferguson et al., 2026	Clinical Considerations for Hormone Therapy in Idiopathic Primary Ovarian Insufficiency	No primary care role or identifiable care pathway	PCC context not met: POI hormone-therapy commentary is not directed to primary care and does not define a primary-care role or pathway.

Note. For the corrective 25-report full-text set (24 reports promoted from the second pass plus Mittal 2014, reconsidered as described above), the reviewers, assessing independently, agreed on 21 (84.0%; Cohen's κ = 0.68). The four disagreements were resolved by the third reviewer (C.A.H.) acting as arbiter: one report was included and three were excluded. Agreement statistics refer to independent decisions before arbitration. After reconciliation, one included report (Dempsey 2020) was excluded as a conference abstract without full text, and two (O'Sullivan 2016; Park 2010) were reclassified as not retrieved (Table S2C).

Table S2C. Reports sought but not retrieved (*n* = 13).

Report	Title	Identifier	Retrieval attempts	Reason
Park et al., 2010	Premature menopause linked to CVD and osteoporosis.	No DOI	Institutional access (University of Oradea); open-access repositories and web search, October 2026	Subscription journal; retrieval of the full text could not be confirmed after reconciliation
Nelson, 2011	One world, one woman: A transformational leader's approach to primary ovarian insufficiency	DOI 10.1097/gme.0b013e318213f250	Institutional access (University of Oradea); open-access repositories and web search, October 2026	Full text not obtained by the review team
Gibson-Helm et al., 2014	Symptoms, health behavior and understanding of menopause therapy in women with premature menopause	DOI 10.3109/13697137.2014.913284	Institutional access (University of Oradea); open-access repositories and web search, October 2026	Full text not obtained by the review team
O'Sullivan et al., 2016	Managing debilitating menopausal symptoms.	No DOI	Institutional access (University of Oradea); open-access repositories and web search, October 2026	Subscription journal; retrieval of the full text could not be confirmed after reconciliation
Hipp et al., 2016	Reproductive and gynecologic care of women with fragile X primary ovarian insufficiency (FXPOI).	DOI 10.1097/GME.0000000000000658	Institutional access (University of Oradea); open-access repositories and web search, October 2026	Full text not obtained by the review team

Report	Title	Identifier	Retrieval attempts	Reason
Singer, 2019	Managing the psychological sequelae of POI	DOI 10.1177/2053369119874640	Institutional access (University of Oradea); open-access repositories and web search, October 2026	Full text not obtained by the review team
Lambrinoudaki et al., 2022	Menopause, wellbeing and health: A care pathway from the European Menopause and Andropause Society	DOI 10.1016/j.maturitas.2022.04.008	Institutional access (University of Oradea); open-access repositories and web search, October 2026	Full text not obtained by the review team
van Zwol-Janssens et al., 2024	Introducing a value-based healthcare approach for women with premature ovarian insufficiency (POI): Recommendations for patient-centered outcomes in clinical practice	DOI 10.1016/j.maturitas.2024.107971	Institutional access (University of Oradea); open-access repositories and web search, October 2026	Full text not obtained by the review team
Panay et al., 2024	Updated premature ovarian insufficiency guideline	DOI 10.1080/13697137.2024.2408922	Institutional access (University of Oradea); open-access repositories and web search, October 2026	Full text not obtained by the review team
Fadem et al., 2026	"I had to work it out for myself": opportunities for primary care to fill a void for early-onset breast cancer survivors	DOI 10.1007/s11764-026-02001-9	Institutional access (University of Oradea); open-access repositories and web search, October 2026	Not available through institutional subscription or open access
Zheng et al., 2026	Telemedicine follow-up improves early detection of ovarian dysfunction after uterine artery embolization: a retrospective cohort study	DOI 10.1186/s12905-026-04477-0	Institutional access (University of Oradea); open-access repositories and web search, October 2026	Full text not obtained by the review team
Hira et al., 2026	Mapping menopause: a rapid review exploring complex menopause care	DOI 10.1097/GME.0000000000002773	Institutional access (University of Oradea); open-access repositories and web search, October 2026	Not available through institutional subscription or open access
Zhang et al., 2026	Interpretation of the guidelines for the prevention, treatment and clinical management of iatrogenic premature ovarian insufficiency in clinical practice; [医源性早发性卵巢功能不全防治与临床管理指南解读]	DOI 10.3969/j.issn.1006-7795.2026.04.001	Institutional access (University of Oradea); open-access repositories and web search, October 2026	Full text not obtained by the review team

Note. Retrieval was attempted through the institutional library access of the University of Oradea and through searches for open-access versions (PubMed Central, publisher websites and web search) in October 2026. No article was purchased, and authors were not contacted, as stated in the registered protocol. O'Sullivan 2016 and Park 2010 had been included at full-text reconciliation; they were reclassified as not retrieved because retrieval of the full text could not be confirmed.

Table S3. Reports identified through other methods (*n* = 14): seven included and seven excluded after full-text assessment. None was present in the three database exports (checked by DOI and normalised title).

Source	Citation (short)	Identification route	Evidence type	Final status
Alzubaidi 2002 [10]	Meeting the needs of young women with secondary amenorrhea and spontaneous premature ovarian failure. Obstet Gynecol	Reference list of an included source (Davies and Cartwright 2012)	Original	Included
Deeks 2011 [11]	Premature menopause: a comprehensive understanding of psychosocial aspects. Climacteric	Reference list of an included source (Yeganeh et al. 2020)	Original	Included
Yeganeh 2019 [29]	Menopause guideline appraisal and algorithm development for premature ovarian insufficiency. Maturitas	Reference list of an included source (Yeganeh et al. 2020)	Secondary	Included

Source	Citation (short)	Identification route	Evidence type	Final status
Hammond 2025 [32]	Women's experiences of premature ovarian insufficiency: a thematic synthesis. Psychol Health	Targeted web search (institutional repository)	Secondary	Included
Tedajo-Tsambou 2024 [56]	A retrospective audit of general practitioner's referrals to Guy's and St Thomas' specialist menopause clinic between 2021 and 2022. Post Reprod Health	Targeted web search	Original	Included
Malick 2026 [62]	Use of hormone therapy in patients with premature ovarian insufficiency in tertiary hospitals in Saudi Arabia. Menopause	Targeted web search	Original	Included
Aubin 2026 [60]	Women's experience of premature ovarian insufficiency (POI) diagnosis: update on actual practices and women's follow-up (EMPOIHER study). Menopause	Trial registry (ClinicalTrials.gov NCT06228547), then published report	Original	Included
Tucker 2016	Premature ovarian insufficiency: new perspectives on genetic cause and phenotypic spectrum. Endocr Rev	Reference lists and hand-searching	Secondary	Excluded: genetic causes of POI, no care-pathway content
Webber 2017	HRT for women with premature ovarian insufficiency: a comprehensive review. Hum Reprod Open	Reference lists and hand-searching	Secondary	Excluded: HRT review, no primary care content
Luisi 2015	Premature ovarian insufficiency: from pathogenesis to clinical management. J Endocrinol Invest	Reference lists and hand-searching	Secondary	Excluded: no primary care content
Podfigurna-Stopa 2016	Premature ovarian insufficiency: the context of long-term effects. J Endocrinol Invest	Reference lists and hand-searching	Secondary	Excluded: no care-pathway content
Sullivan 2016	Hormone replacement therapy in young women with primary ovarian insufficiency and early menopause. Fertil Steril	Reference lists and hand-searching	Secondary	Excluded: HRT review, no primary care content
Rudnicka 2018	Premature ovarian insufficiency: aetiopathology, epidemiology, and diagnostic evaluation. Menopause Rev	Reference lists and hand-searching	Secondary	Excluded: no primary care or pathway content
Touraine 2024	Premature ovarian insufficiency. Nat Rev Dis Primers	Reference lists and hand-searching	Secondary	Excluded: disease primer, no primary care or pathway content

Table S4. Data-charting table for the 53 included sources. Reference numbers correspond to the main manuscript. Funding and conflict-of-interest entries reproduce the declarations published with each source: "Not reported" means that no statement was identified in the full text and does not imply absence of funding or conflicts; "Not verifiable" means that the declaration fields were not accessible to the review team.

Source	Ref.	Country	Evidence type	Design / population	Care-continuum stage(s)	Primary care role	Main finding relevant to primary care	Identified via	Funding (as declared)	Conflicts of interest (as declared)
Davies 2012	[21]	United Kingdom	Secondary/prescriptive	Expert review (clinical question)	Recognition; Diagnostic delay; Referral/pathways; Follow-up/HT	Implied	Patients see three or more professionals before diagnosis; annual review, DXA every 3-5 years, dedicated POI services	Database	Not reported: No explicit funding statement identified in the uploaded full text.	Not reported: No explicit conflict-of-interest declaration identified in the uploaded full text.
Klein 2013	[22]	USA	Secondary/prescriptive	Review for family physicians (amenorrhoea)	Recognition; Follow-up/HT	Explicit	Structured primary care work-up for amenorrhoea including recognition of ovarian insufficiency and long-term treatment	Database	Not reported: No source-level funding statement identified.	None declared: American Family Physician author disclosure: no relevant financial affiliations identified.
Gordon 2015	[23]	USA	Secondary/prescriptive	Narrative review (adolescents)	Recognition; Diagnostic delay; Referral/pathways	Explicit	Delay because irregular menses assumed common in adolescents; integrated care coordinated by a primary care clinician	Database	No funding: Financial support and sponsorship: None.	None declared: The authors reported no conflicts of interest.
van Dorp 2016	[24]	International	Secondary/prescriptive	Harmonised surveillance guideline	Referral/pathways; Follow-up/HT	Implied	Surveillance for POI in cancer survivors exposed to alkylating agents or pelvic radiotherapy, with FSH/oestradiol assessment and referral for HT	Database	Declared funding: Support included KOCR; Dutch Cancer Society (UVA 2011-4938); St Jude CORE grant CA21765/ALSAC; and EU FP7 PanCareSurFup (257505), among...	None declared: No relevant relationships were disclosed in the article's author disclosure information.
Lukman 2017	[14]	United Kingdom	Secondary/prescriptive	Textbook chapter for primary care	Recognition; Follow-up/HT	Explicit	Consider POI after three months of amenorrhoea; HRT or COCP until at least 51; risks quoted for older women do not apply; 5-10% pregnancy rate	Database	Not reported: No explicit funding statement identified in the uploaded book chapter.	Not reported: No explicit conflict-of-interest statement identified in the uploaded book chapter.

Source	Ref.	Country	Evidence type	Design / population	Care-continuum stage(s)	Primary care role	Main finding relevant to primary care	Identified via	Funding (as declared)	Conflicts of interest (as declared)
Nguyen 2017	[15]	Australia	Secondary/prescriptive	Review for GPs with index case	Recognition; Referral/pathways; Follow-up/HT	Explicit	GPs play an important role in evaluation and long-term management; two FSH values 4-6 weeks apart; HRT to natural menopause; contraception; multidisciplinary management	Database	Not reported: No article-specific funding source was stated. Amanda Vincent disclosed research grants from Amgen outside the submitted work.	Declared COI: Amanda Vincent disclosed personal fees from Novo Nordisk and research grants/personal fees from Amgen outside the submitted work; Hanh...
Machura 2018	[25]	Poland	Secondary/prescriptive	Narrative review	Follow-up/HT	Not applicable	The narrative review reports that approximately 40% discontinue HRT within the first year; this is a secondary claim, not a primary estimate generated by this source. Multidisciplinary care is recommended.	Database	Not reported: No explicit funding statement identified in the uploaded full text.	None declared: The authors reported no conflict of interest.
Newson 2018	[26]	United Kingdom	Secondary/prescriptive	Editorial by GPs	Recognition; Diagnostic delay; Follow-up/HT; Knowledge/attitudes	Explicit	POI underdiagnosed and undertreated in primary care; many GPs not confident diagnosing it; women told they are too young to be menopausal	Database	Not reported: No explicit funding statement identified in the uploaded editorial.	Not reported: No explicit conflict-of-interest statement identified in the uploaded editorial.
Kiriakova 2019	[27]	Australia	Secondary/prescriptive	Guideline appraisal (AGREE II), 16 guidelines	Follow-up/HT	Not applicable	Variable guideline quality; bone health algorithm developed	Database	Declared funding: Supported by an Osteoporosis Australia–ANZBMS 2016 grant awarded to A.J. Vincent.	Declared COI: A.J. Vincent served on the editorial board of Climacteric, one of the journals represented among appraised guidelines; all other authors...
Klein 2019	[28]	USA	Secondary/prescriptive	Review for family physicians (amenorrhoea)	Recognition; Follow-up/HT	Explicit	POI in the routine differential diagnosis of amenorrhoea; first-line laboratory assessment; hormone replacement until the usual age of menopause	Database	Not reported: No source-level funding statement identified.	None declared: American Family Physician author disclosure: no relevant financial affiliations identified.

Source	Ref.	Country	Evidence type	Design / population	Care-continuum stage(s)	Primary care role	Main finding relevant to primary care	Identified via	Funding (as declared)	Conflicts of interest (as declared)
Yeganeh 2019	[29]	Australia	Secondary/prescriptive	Guideline appraisal, 22 guidelines	Recognition; Follow-up/HT	Not applicable	Only 2 of 22 menopause guidelines high quality (NICE, ESHRE); EM/POI algorithms developed	Other methods	Declared funding: Australian NHMRC Partnership Grant APP1116008.	Declared COI: Jacqueline Boyle disclosed membership of the RANZCOG Women's Health Committee; Amanda Vincent disclosed membership of the Climacteric...
Jones 2020 (SRM)	[30]	Australia	Secondary/prescriptive	Systematic search and narrative review	Referral/pathways	Not applicable	No model of care recommended for POI; 42 components in six themes	Database	Declared author support / no direct study funding: A.R.J. was supported by NHMRC postgraduate scholarship 1169192 and an Avant Doctor in Training Research Scholarship; the study stated...	None declared: The authors declared no known conflict of interest.
Lambrinoudaki 2021	[13]	International (EMAS/IMS)	Secondary/prescriptive	Expert review for primary care	Recognition; Diagnostic delay; Referral/pathways; Follow-up/HT; Knowledge/attitudes	Explicit	Diagnosis and treatment often delayed; need for education of the broad medical community; diagnostic approach, investigations, HRT and follow-up for the primary care physician	Database	No funding: No funding was received for the preparation of the article.	Declared COI: M.A. Lumsden disclosed advisory work for Novo Nordisk and Roche Diagnostics; N. Panay disclosed lecture/advisory relationships with...
Jayasena 2024	[31]	United Kingdom	Secondary/prescriptive	Society for Endocrinology guideline (female hypogonadism)	Recognition; Referral/pathways; Follow-up/HT	Explicit	Cross-specialty diagnostic steps and referral thresholds for female hypogonadism, including POI; developed with primary care input	Database	Author/institutional support reported: The guideline article disclosed author research support including NIHR fellowships/HTA awards and related institutional support; no...	Declared COI: Several authors disclosed grants, speaker fees or sponsorship/industry relationships; declarations were reported in the guideline.

Source	Ref.	Country	Evidence type	Design / population	Care-continuum stage(s)	Primary care role	Main finding relevant to primary care	Identified via	Funding (as declared)	Conflicts of interest (as declared)
Panay 2024	[1]	International	Secondary/prescriptive	Evidence-based guideline	Recognition; Referral/pathways; Follow-up/HT	Explicit	145 recommendations; diagnosis on cycle disturbance of 4 months plus a single FSH above 25 IU/L; AMH confirmatory only and noted as not universally available in primary care; HT until at least average age of menopause	Database	Declared funding: Guideline development funded by ESHRE, ASRM, CRE-WHiRL and IMS, covering meetings, literature searches and dissemination;...	Declared COI: Multiple guideline-group members disclosed grants, consultancy, honoraria, travel support, board/advisory roles and other relationships;...
Hammond 2025	[32]	United Kingdom	Secondary/prescriptive	Thematic synthesis, 10 qualitative studies	Diagnostic delay; Follow-up/HT	Implied	Difficulty accessing support before and after diagnosis; HCP training needed	Other methods	No funding: No funding associated with the study was reported.	None declared: No competing interests were reported.
Sugianto 2025	[33]	Singapore	Secondary/prescriptive	Practical toolkit (workgroup including family medicine)	Recognition; Referral/pathways; Follow-up/HT	Explicit	Diagnosis, HT prescribing and counselling, referral and long-term management of POI	Database	Not reported: No explicit source-level funding statement identified in the accessible article.	None declared: Authors stated no affiliations or financial involvement with a commercial organization having a direct financial interest in the subject...
Benedetto 2026	[34]	International	Secondary/prescriptive	Joint FIGO/IMS position paper	Recognition; Referral/pathways; Follow-up/HT	Implied	Integrated life-course pathway from early identification and risk assessment to HT, reassessment and longitudinal follow-up; GPs named among clinicians encountering POI	Database	Not reported: No explicit funding statement identified in the open publisher full text.	Declared COI: Multiple authors disclosed advisory, lecture, society leadership and/or industry relationships; S.S. Khadilkar and F. Frezet declared no...
Klein 2026	[35]	USA	Secondary/prescriptive	Review for family physicians (amenorrhoea)	Recognition; Follow-up/HT	Explicit	Updated practical amenorrhoea work-up; POI as a diagnosis requiring hormone therapy and chronic risk management	Database	Not reported: No source-level funding statement identified.	None declared: American Family Physician author disclosure reported no relevant financial relationships.
Nelson 2026	[36]	USA	Secondary/prescriptive	Clinical framework	Recognition	Explicit	Menstrual cycle as a vital sign in primary care, POI as sentinel condition	Database	No funding: The author reported receiving no financial support for the research, authorship and/or publication of the article.	Declared COI: The author disclosed employment by the Mary Elizabeth Conover Foundation and an editorial-board role with Frontiers.

Source	Ref.	Country	Evidence type	Design / population	Care-continuum stage(s)	Primary care role	Main finding relevant to primary care	Identified via	Funding (as declared)	Conflicts of interest (as declared)
Vincent 2026	[9]	Australia	Secondary/prescriptive	Guideline summary for general practitioners	Recognition; Referral/pathways; Follow-up/HT	Explicit	GP roles: early identification, diagnosis, initial chronic disease risk assessment, coordination of referrals and long-term follow-up	Database	Declared related funding / no author remuneration: Development of the underlying POI guideline included funding from the NHMRC Centre of Excellence Women's Health in Reproductive Life...	Declared COI: A.J. Vincent disclosed a trial with medication/placebo supplied by Lawley Pharmaceuticals, IMS board/travel support, personal fees from...
Alzubaidi 2002	[10]	USA	Original empirical	Patient survey (*n* = 50)	Diagnostic delay	Implied	More than half saw three or more clinicians before diagnosis; a quarter waited over five years	Other methods	Not verifiable: No funding declaration was available in the accessible abstract/metadata; full declaration could not be confirmed.	Not verifiable: No COI declaration was available in the accessible abstract/metadata; full declaration could not be confirmed.
Deeks 2011	[11]	Australia	Original empirical	Patient survey	Diagnostic delay	Implied	Delay up to 1.9 years; GP a less expected information source than gynaecologist or internet	Other methods	Declared author support / no project funding for 3 authors: Deeks, Gibson-Helm and Vincent stated they received no funding for the project; H. Teede was an NHMRC research fellowship holder.	Declared COI: A. Vincent disclosed membership of the Climacteric editorial board.
Singer 2011	[38]	United Kingdom	Original empirical	Survey, two clinics and self-help website	Diagnostic delay; Follow-up/HT	Explicit	30% diagnosed within 6 months, 22% took up to 3 years; sensitive handling often lacking in primary care	Database	Declared funding: Seedcorn funding from the British Association for Counselling and Psychotherapy (BACP).	None declared: The authors reported no conflict of interests.
Cartwright 2012	[39]	United Kingdom	Original empirical	Service evaluation, tertiary POF clinic (176 new referrals)	Referral/pathways; Follow-up/HT	Explicit	23% seen within 4 months of GP diagnosis, 33% after more than a year; 25% of new appointments missed; GPs advising women to stop HRT	Database	Not reported: No explicit study-funding declaration identified in the uploaded full text.	None declared: The authors reported no conflicts of interest.

Source	Ref.	Country	Evidence type	Design / population	Care-continuum stage(s)	Primary care role	Main finding relevant to primary care	Identified via	Funding (as declared)	Conflicts of interest (as declared)
Little 2014	[40]	Australia	Original empirical	Case series, 3 adolescents seen in general practice	Recognition	Explicit	All three had been prescribed the combined oral contraceptive pill for menstrual irregularity before the investigations that led to diagnosis	Database	No funding: No financial support was reported for the article.	None declared: No conflicts of interest were reported.
Mittal 2014	[20]	United Kingdom	Original empirical	Retrospective review, 223 consecutive referrals to a tertiary POI clinic	Diagnostic delay; Referral/pathways; Follow-up/HT	Not studied directly	24.1% (19/79) presented within 12 months of symptom onset; 58.7% on HRT; 69.5% had DXA; reduced BMD more likely with longer delay	Database	Not verifiable: Funding declaration was not retrievable from the accessible publisher abstract/metadata.	Not verifiable: COI declaration was not retrievable from the accessible publisher abstract/metadata.
Nekhlyudov 2014	[41]	USA	Original empirical	National survey, 1130 oncologists and 1072 PCPs	Knowledge/attitudes	Explicit	15% of PCPs vs 71% of oncologists aware of premature menopause after cyclophosphamide	Database	Declared funding: Supported by National Cancer Institute Grant HHSN261201000316C and intramural research funding from the American Cancer Society.	None declared: The authors indicated no potential conflicts of interest.
Yeganeh 2017	[42]	Australia	Original empirical	Online survey, 745 HPs	Knowledge/attitudes	Explicit	COCP preferred for premature menopause (52%); 58% would continue to 51; duration varied by specialty	Database	No funding: Source of funding: Nil.	Declared COI: Amanda Vincent disclosed Climacteric editorial-board membership and President-Elect role in the Australasian Menopause Society; no other...
Kanj 2018	[43]	USA	Original empirical	Chart review, 50 adolescents and young adults	Recognition; Follow-up/HT	Not studied directly	72% karyotyped, 28% adrenal antibodies, 64% DXA; only 4% saw mental health support	Database	Not reported: No explicit funding statement identified in the uploaded full text.	None declared: The authors reported no proprietary/commercial interests and no conflicts of interest.
Neill 2018	[44]	United Kingdom	Original empirical	Referral audit, 50 GP letters	Referral/pathways	Explicit	POI in 2 of 50 GP referrals; national referral guidance vague; local pre-referral advice developed	Database	No funding: The author(s) received no financial support for the research, authorship and/or publication.	None declared: The author(s) declared no potential conflict of interest.

Source	Ref.	Country	Evidence type	Design / population	Care-continuum stage(s)	Primary care role	Main finding relevant to primary care	Identified via	Funding (as declared)	Conflicts of interest (as declared)
Richardson 2018	[45]	United Kingdom	Original empirical	Multidisciplinary review of practice, 103 women	Recognition; Referral/pathways; Follow-up/HT	Not studied directly	Karyotype 40.6%, FMR1 7.4%, DXA 35.9%; HRT discussed 76%, psychological support 25%; wide variation by specialty	Database	No funding: The author(s) received no financial support for the research, authorship and/or publication.	None declared: The author(s) declared no potential conflicts of interest.
Demayo 2019	[46]	Argentina	Original empirical	Multicentre registry, 302 women	Recognition; Diagnostic delay	Not studied directly	About half had oligomenorrhoea for years before diagnosis; 27% work-up incomplete	Database	Not verifiable: No funding declaration was identified in the accessible article metadata/abstract during this audit.	Not verifiable: No COI declaration was identified in the accessible article metadata/abstract during this audit.
Hoyos-Martinez 2019	[47]	USA	Original empirical	Case series, 7 idiopathic adolescents	Recognition; Diagnostic delay; Referral/pathways	Implied	Mean 3.8 years from menarche to diagnosis; FMR1 tested in 40%; 29% referred for reproductive counselling	Database	No funding: Research funding: None declared.	None declared: Employment/leadership: none; honorarium: none; no relevant competing interest was reported.
Johnston-Ataata 2020	[48]	Australia	Original empirical	Narrative interviews, 30 women	Diagnostic delay	Implied	Diagnosis a complex, changeable process negotiated across providers	Database	Declared funding: NHMRC Partnership Grant APP1116008 and National Breast Cancer Foundation grant PE-16-002.	Not reported: No explicit conflict-of-interest statement was identified in the accessible article record/full-text information reviewed.
Jones 2020 (Arch Osteoporos)	[49]	Australia	Original empirical	Online surveys, 688 GPs and 316 women	Follow-up/HT; Knowledge/attitudes	Explicit	GP knowledge 9.1/13; 41% reported barriers to care, varying by location	Database	Declared funding: A.R.J.: NHMRC postgraduate scholarship and Avant Doctor in Training Research Scholarship; J.B.: NHMRC Career Development fellowship;...	None declared: Conflict of interest: None.
Yeganeh 2020 (Menopause)	[50]	Australia	Original empirical	Co-design, 263 women, pilot	Follow-up/HT	Implied	Communication difficulties on sexual (50.6%), urogenital (43%) and psychological (41%) issues; question prompt list feasible	Database	Declared funding: NHMRC Partnership Grant APP1116008.	None declared: Financial disclosure/conflicts of interest: None reported.

Source	Ref.	Country	Evidence type	Design / population	Care-continuum stage(s)	Primary care role	Main finding relevant to primary care	Identified via	Funding (as declared)	Conflicts of interest (as declared)
Yeganeh 2020 (SRM)	[51]	Australia	Original empirical	Mixed-methods co-design	Recognition; Knowledge/attitudes	Explicit	Practitioners incl. GPs call for faster, more empathetic diagnosis; digital resources rated useful by 80%	Database	Declared funding: NHMRC Partnership Grant APP1116008.	None declared: Conflict of interest: None declared.
Manley 2021	[52]	United Kingdom	Original empirical	Service evaluation, tertiary and district clinics	Referral/pathways	Explicit	Most referrals from primary care; 30% of new referrals POI; 30% seen after 18 weeks at tertiary centre	Database	Not verifiable: Funding declaration was not visible in the accessible restricted publisher record.	Not verifiable: COI declaration was not visible in the accessible restricted publisher record.
Minis 2022	[12]	USA	Original empirical	Case series, safety-net clinic, 19 of 524 patients	Diagnostic delay; Referral/pathways; Follow-up/HT	Implied	Median 48 months from symptoms to diagnosis; of 9 diagnosed before referral only 4 on HT	Database	No external funding: The research received no external funding.	None declared: The authors declared no conflicts of interest.
Beshyah 2023	[53]	Middle East and Africa	Original empirical	Survey, 254 physicians (36% family medicine)	Knowledge/attitudes	Explicit	Obstetricians more likely to treat premature ovarian failure to age 50 (71.8%)	Database	No funding: No funding was received for the study.	None declared: The authors declared that no competing interests exist.
Poteet 2023	[54]	USA	Original empirical	Qualitative interviews, 24 women with FXPOI	Diagnostic delay	Implied	Women advocated for their own diagnosis across multiple providers	Database	Declared funding: National Fragile X Foundation; Georgia Association of Genetic Counselors; NICHD/NINDS support to the National Fragile X Center...	None declared: The authors declared no competing interests.
Copp 2024	[55]	Australia	Original empirical	Nationwide survey, 362 clinicians	Recognition; Knowledge/attitudes	Explicit	GPs order AMH less often, mostly at patient request; 22% believe it predicts premature menopause	Database	Declared funding: NHMRC support included Centre for Research Excellence grant 1104136, Program grant 1113532 and Emerging Leader fellowship 2009419.	Declared COI: B.W. Mol, D. Lieberman and K. Hammarberg disclosed specified consultancy/travel or industry relationships; other authors reported none...
Tedajo-Tsambou 2024	[56]	United Kingdom	Original empirical	Audit, 50 GP referrals to tertiary clinic	Referral/pathways	Explicit	POI second most common referral reason (20%); one sixth manageable by other routes	Other methods	No funding: The author(s) received no financial support for the research, authorship and/or publication.	None declared: The author(s) declared no potential conflicts of interest.

Source	Ref.	Country	Evidence type	Design / population	Care-continuum stage(s)	Primary care role	Main finding relevant to primary care	Identified via	Funding (as declared)	Conflicts of interest (as declared)
Bakhsh 2025	[57]	Saudi Arabia	Original empirical	Retrospective cohort	Diagnostic delay; Follow-up/HT	Not studied directly	Delayed diagnosis, absence of HT and lack of fertility counselling were independently associated with higher odds of psychological distress	Database	No external funding: The study reported no external research funding; the APC was funded by Princess Nourah bint Abdulrahman University.	None declared: The author declared no conflicts of interest.
Csehely 2025	[58]	Hungary	Original empirical	Retrospective cohort, 125 women	Diagnostic delay	Not studied directly	Time to diagnosis inversely correlated with BMD (r = -0.225)	Database	No external funding: This research received no external funding.	None declared: The authors declared no conflicts of interest.
Jeevananthan 2025	[59]	USA	Original empirical	Provider survey, 201 (102 PCPs)	Follow-up/HT; Knowledge/attitudes	Explicit	45% of PCPs vs 82-86% of specialists always consider HT appropriate in POI	Database	No specific study grant / author support: The research received no specific grant; the corresponding author's postdoctoral fellowship was supported by NIH T32 DK007012 and A.S.A....	None declared: The authors declared no conflicts of interest.
Aubin 2026	[60]	France	Original empirical	Multicentre cohort, 101 women with spontaneous POI (EMPOIHER)	Follow-up/HT	Implied	63.4% on MHT at inclusion; 70% sought information from the internet or another practitioner; psychological support offered to 19.8%	Other methods	Not reported: No explicit funding/support statement was identified in the accessible PubMed record; the trial sponsor was Bordeaux University Hospital.	Declared COI: E. Begon disclosed a current financial relationship with Gedeon Richter; all other authors reported nothing to disclose.
Cavadias 2026	[37]	France	Secondary/prescriptive	Narrative review reporting descriptive data from a cardio-gynaecological pathway (52 women with POI)	Referral/pathways; Follow-up/HT	Implied	43.1% of women with POI received a new cardiovascular risk diagnosis; letter to GP as pathway output	Database	No external funding: No funding from an external source was received for the study/article.	None declared: The authors declared no competing interest.
Langhe 2026	[16]	United Arab Emirates	Original empirical	Physician survey, 411	Recognition; Follow-up/HT; Knowledge/attitudes	Explicit	20.4% applied diagnostic criteria; HT initiated by 29.2% of PCPs vs 65.2% of specialists	Database	No funding: No financial support was reported for the study/article.	None declared: The authors declared no competing interests.

Source	Ref.	Country	Evidence type	Design / population	Care-continuum stage(s)	Primary care role	Main finding relevant to primary care	Identified via	Funding (as declared)	Conflicts of interest (as declared)
Liu 2026	[61]	China	Original empirical	Community KAP survey	Recognition	Implied	Constrained knowledge of POI among women of childbearing age	Database	No funding: The authors declared that financial support was not received for the work and/or its publication.	None declared: The authors declared the absence of commercial or financial relationships that could be construed as a potential conflict of interest.
Malick 2026	[62]	Saudi Arabia	Original empirical	Chart review, three tertiary hospitals	Follow-up/HT	Not studied directly	Only 36% of women with POI received HT	Other methods	No funding: Funding/Support: None reported.	None declared: Financial disclosure/conflicts of interest: None reported.
Pisana 2026	[63]	Australia, UK, USA	Original empirical	Online qualitative, 191 women	Diagnostic delay; Follow-up/HT	Explicit	Provider dismissal and limited awareness; care cliff at midlife; call for primary care education	Database	Declared funding: Supported by the Commonwealth through an Australian Government Research Training Program Scholarship (DOI 10.82133/C42F-K220).	None declared: The authors declared no competing interest.

Table S5. Coding framework, operational definitions and screening decision rules.

Variable / rule	Operational definition	Examples and decision rules
Evidence type: original empirical	Source reports data collected by its authors from patients, clinicians, records or registries.	Surveys, cohorts, chart reviews, audits, service evaluations, case series, interview studies.
Evidence type: secondary/prescriptive	Source summarises or prescribes care without collecting new data, classified according to the design stated by the publication. A narrative review that also reports descriptive pathway data is classified as secondary; its descriptive data are used in the synthesis without counting it as an original empirical source.	Guidelines, consensus and position papers, reviews, guideline appraisals, editorials, textbook chapters.
Stage 1: Recognition and initial evaluation	Content on presenting features, when to suspect POI, diagnostic criteria, first-line tests or initial aetiological work-up.	Diagnostic thresholds; tests at first evaluation; recognition of symptoms.
Stage 2: Diagnostic delay	Measures or describes time to diagnosis, number of clinicians consulted before diagnosis, or delayed or dismissed diagnosis.	Months to diagnosis or presentation; dismissal; delay related to outcomes.
Stage 3: Referral and interdisciplinary pathways	Content on referral source, timing or appropriateness, variation between specialties, multidisciplinary services, care pathways or models of care.	Referral audits; time from GP diagnosis to clinic; integrated pathways.
Stage 4: Long-term follow-up and hormone therapy	Content on hormone therapy initiation, adherence or persistence; bone and cardiovascular surveillance; ongoing information and support.	HT uptake; discontinuation; DXA; survivorship surveillance.
Stage 5: Physician knowledge and attitudes	Measures or describes clinicians' knowledge, attitudes or practice regarding POI.	Physician surveys.
Multiple stages	A source is assigned to every stage for which it contains substantive content; stage counts are not mutually exclusive.	
Primary care role: explicit	General practitioners, family physicians or primary care physicians are a studied population or the stated audience or setting, or referral from or to primary care is measured.	GP surveys; GP referral audits; reviews written for GPs.
Primary care role: implied	Primary care is not the studied population or audience, but its role is identifiable from the referral or care pathway described.	Patient surveys on diagnostic journeys; pathways naming GPs.
Primary care role: not studied directly	Specialist-setting empirical study that does not study primary care but reports a care-pathway outcome (time to diagnosis or presentation, referral, or continuity of treatment and follow-up).	Tertiary cohorts reporting delay or hormone therapy uptake.
Primary care role: not applicable	Secondary source in which primary care involvement is not a meaningful variable.	Guideline appraisals; frameworks.
R1	A record without an abstract cannot be excluded at the abstract stage; it proceeds to full-text assessment.	
R2	Sources on amenorrhoea, menopause, hypogonadism or cancer survivorship in general are included only if they contain distinct POI-specific content on recognition, referral or follow-up.	
R3	Duplicate, co-published or superseded versions of the same source are counted once; the most complete or current version is retained.	
R4	Case series of two or more patients are eligible; single-patient case reports are excluded.	
R5	Sources on early menopause (40 to 45 years) only, or on surgical menopause without separable POI data, are excluded.	
S1	Empirical clinician surveys restricted to specialists, without a primary care subgroup or a referral, shared-care or pathway outcome, are excluded under the registered context criterion.	

Table S6. Evidence map: contribution of each included source to the five care-continuum stages.

Source [ref.]	Recognition	Diagnostic delay	Referral/pathways	Follow-up/HT	Knowledge/attitudes
Davies 2012 [21]	✓	✓	✓	✓	

Source [ref.]	Recognition	Diagnostic delay	Referral/pathways	Follow-up/HT	Knowledge/attitudes
Klein 2013 [22]	✓			✓	
Gordon 2015 [23]	✓	✓	✓		
van Dorp 2016 [24]			✓	✓	
Lukman 2017 [14]	✓			✓	
Nguyen 2017 [15]	✓		✓	✓	
Machura 2018 [25]				✓	
Newson 2018 [26]	✓	✓		✓	✓
Kiriakova 2019 [27]				✓	
Klein 2019 [28]	✓			✓	
Yeganeh 2019 [29]	✓			✓	
Jones 2020 (SRM) [30]			✓		
Lambrinoudaki 2021 [13]	✓	✓	✓	✓	✓
Jayasena 2024 [31]	✓		✓	✓	
Panay 2024 [1]	✓		✓	✓	
Hammond 2025 [32]		✓		✓	
Sugianto 2025 [33]	✓		✓	✓	
Benedetto 2026 [34]	✓		✓	✓	
Klein 2026 [35]	✓			✓	
Nelson 2026 [36]	✓				
Vincent 2026 [9]	✓		✓	✓	
Alzubaidi 2002 [10]		✓			
Deeks 2011 [11]		✓			
Singer 2011 [38]		✓		✓	
Cartwright 2012 [39]			✓	✓	
Little 2014 [40]	✓				
Mittal 2014 [20]		✓	✓	✓	
Nekhlyudov 2014 [41]					✓
Yeganeh 2017 [42]					✓
Kanj 2018 [43]	✓			✓	
Neill 2018 [44]			✓		
Richardson 2018 [45]	✓		✓	✓	
Demayo 2019 [46]	✓	✓			

Source [ref.]	Recognition	Diagnostic delay	Referral/pathways	Follow-up/HT	Knowledge/attitudes
Hoyos-Martinez 2019 [47]	✓	✓	✓		
Johnston-Ataata 2020 [48]		✓			
Jones 2020 (Arch Osteoporos) [49]				✓	✓
Yeganeh 2020 (Menopause) [50]				✓	
Yeganeh 2020 (SRM) [51]	✓				✓
Manley 2021 [52]			✓		
Minis 2022 [12]		✓	✓	✓	
Beshyah 2023 [53]					✓
Poteet 2023 [54]		✓			
Copp 2024 [55]	✓				✓
Tedajo-Tsambou 2024 [56]			✓		
Bakhsh 2025 [57]		✓		✓	
Csehely 2025 [58]		✓			
Jeevananthan 2025 [59]				✓	✓
Aubin 2026 [60]				✓	
Cavadias 2026 [37]			✓	✓	
Langhe 2026 [16]	✓			✓	✓
Liu 2026 [61]	✓				
Malick 2026 [62]				✓	
Pisana 2026 [63]		✓		✓	
Total	25	17	20	33	10

Table S7. Deduplication procedure.

Step	Description
Export formats	PubMed (PubMed format, with abstracts); Scopus (CSV, with abstracts and keywords); Web of Science (plain text, full record).
Order of criteria	1) DOI (exact match, case-insensitive); 2) PMID; 3) normalised title (lowercase, letters and digits only).
Sequence	Web of Science records formed the base set; PubMed records were compared against it, then Scopus records against the combined set.
Duplicates removed (*n* = 521)	Within Web of Science: 2 (same article indexed twice, matched by normalised title); PubMed records already in Web of Science: 194; Scopus records already in Web of Science or PubMed: 325. 1327 records minus 521 duplicates = 806 unique records.
Co-publications	Co-published versions of the same guidance in different journals (different DOIs) were not merged at deduplication; they were identified at screening or full-text assessment and counted once (R3).
Software	Python 3.12.3 with pandas 3.0.2. The script reproduces these counts from the raw exports and will be deposited in the OSF project linked to the registration upon publication.
